# iMPTCE-Hnetwork: A Multilabel Classifier for Identifying Metabolic Pathway Types of Chemicals and Enzymes with a Heterogeneous Network

**DOI:** 10.1155/2021/6683051

**Published:** 2021-01-04

**Authors:** Yuanyuan Zhu, Bin Hu, Lei Chen, Qi Dai

**Affiliations:** ^1^College of Information Engineering, Shanghai Maritime University, Shanghai 201306, China; ^2^State Key Laboratory of Livestock and Poultry Breeding, Guangdong Public Laboratory of Animal Breeding and Nutrition, Guangdong Provincial Key Laboratory of Animal Breeding and Nutrition, Institute of Animal Science, Guangdong Academy of Agricultural Sciences, Guangzhou 510640, China; ^3^College of Life Sciences, Zhejiang Sci-Tech University, Hangzhou 310018, China

## Abstract

Metabolic pathway is an important type of biological pathways. It produces essential molecules and energies to maintain the life of living organisms. Each metabolic pathway consists of a chain of chemical reactions, which always need enzymes to participate in. Thus, chemicals and enzymes are two major components for each metabolic pathway. Although several metabolic pathways have been uncovered, the metabolic pathway system is still far from complete. Some hidden chemicals or enzymes are not discovered in a certain metabolic pathway. Besides the traditional experiments to detect hidden chemicals or enzymes, an alternative pipeline is to design efficient computational methods. In this study, we proposed a powerful multilabel classifier, called iMPTCE-Hnetwork, to uniformly assign chemicals and enzymes to metabolic pathway types reported in KEGG. Such classifier adopted the embedding features derived from a heterogeneous network, which defined chemicals and enzymes as nodes and the interactions between chemicals and enzymes as edges, through a powerful network embedding algorithm, Mashup. The popular RAndom k-labELsets (RAKEL) algorithm was employed to construct the classifier, which incorporated the support vector machine (polynomial kernel) as the basic classifier. The ten-fold cross-validation results indicated that such a classifier had good performance with accuracy higher than 0.800 and exact match higher than 0.750. Several comparisons were done to indicate the superiority of the iMPTCE-Hnetwork.

## 1. Introduction

Metabolic pathway is an essential type of biological pathways in living organisms. It generates necessary molecules and energies to maintain the life of the organisms [1]. In each metabolic pathway, there are several continuous chemical reactions, which change one molecule to another with the help of some enzymes. Chemicals and enzymes are the two major components in each metabolic pathway. Identification of chemicals and enzymes in each pathway as full as possible is helpful to understand its mechanism. To date, several public databases, such as KEGG [2, 3], provide detailed information of validated metabolic pathways. However, the completeness of each metabolic pathway is still a problem. There still exist undiscovered chemicals or enzymes for some metabolic pathways. The traditional experiment is a solid pipeline to determine novel chemicals or enzymes of metabolic pathways. However, it is always time-consuming and expensive. Thus, it is urgent to design novel approaches to accelerate the detection procedures and decrease the costs.

With the development of computer science and technique, it becomes more and more popular to tackle different biological and medical problems with advanced computational methods. Among them, the machine learning-based method is always an important choice. For the problem addressed in this study, several such methods have been proposed in the recent ten years. Most of them were designed to assign chemicals to corresponding metabolic pathway types. Cai et al. [4] first built a nearest neighbor algorithm- (NNA-) based model to predict metabolic pathway type of chemicals, where chemicals were represented by functional group compositions. Later, Lu et al. [1] improved this model by adopting a more powerful classification algorithm, AdaBoost. These two methods can only deal with chemicals participating in only one metabolic pathway type. In fact, several chemicals can belong to two or more pathway types, inducing the limitation of the above methods. After that, investigators began to design models that can deal with chemicals in multiple metabolic pathway types. Hu et al. [5] gave a computational method with chemical-chemical interaction (CCI) information, which can rank the candidate pathway types for a given chemical. The chemical has the highest probability to participate in the first pathway type, followed by the second pathway type, and so on. Chen et al. [6] adopted the same scheme to list the candidate pathway types. Chemicals were encoded by their molecular fragment features, and a support vector machine (SVM) [7] was adopted to give a score to each pathway type. Although the above methods can process chemicals with multiple pathway types, they were not pure multilabel classifiers because they cannot determine which pathway types are the predicted pathways. Recently, Baranwal et al. [8] presented a powerful multilabel classifier to assign chemicals to multiple pathways, which adopted graph convolutional networks for obtaining the molecular shape features of chemicals. Jia et al. [9] built a multilabel web server, iMPT-FRAKEL, to predict metabolic pathways of chemicals. This web server had wide applications because it only needed the SMILEs strings of chemicals as the input. Besides, some other studies tackled the problem in different ways. Fang and Chen [10] deemed the pairs of chemicals and pathway types as samples. In this case, the multilabel classification problem was transformed into a binary classification problem. Jia et al. [11] extended the above model to an actual metabolic pathway rather than a pathway type. The concept of “similarity” was adopted to extract essential features for each pair of chemical and pathway. Guo et al. [12] constructed a SVM-based model for each pathway type, where chemicals were represented by embedding features extracted from multiple chemical networks. From the above descriptions, we can see that they only tackled one component, chemicals, in metabolic pathways. As for the other component, enzyme, only one study is involved to our knowledge. Gao et al. [13] generalized Hu et al.'s method [5] by employing chemical-protein interaction (CPI) and protein-protein interaction (PPI) information, thereby giving a pathway type rank for a given chemical or enzyme. As mentioned above, such method was not a pure multilabel classifier and it directly used the linkage between chemicals and proteins but not deeply mine the hidden information behind the linkage.

In this study, we adopted the metabolic pathway information reported in KEGG, where chemicals and enzymes are classified into 11 metabolic pathway types. A heterogeneous network was built to organize the chemicals, enzymes, CCI, CPI, and PPI information, where chemicals and enzymes defined nodes and three types of interaction information determined edges. To fully mine deep information in the heterogeneous network, a powerful network embedding algorithm, Mashup [14], was applied to such network. Informative features were obtained for each chemical and enzyme. These features and labels, representing metabolic pathway types, were fed into the RAndom k-labELsets (RAKEL) [15] algorithm to build the classifier, iMPTCE-Hnetwork. SVM (polynomial kernel) [7] was adopted as the basic classifier. The effects of heterogeneous networks and the merits of combining the information of chemicals and enzymes were elaborated. Furthermore, the comparisons of other multilabel classifiers with Binary Relevance (BR) [16], features derived from other network embedding algorithms, or other basic classifiers were done to indicate the superiority of the iMPTCE-Hnetwork.

## 2. Materials and Methods

### 2.1. Materials

The chemicals and enzymes (human) in metabolic pathways were retrieved from the KEGG PATHWAY (https://www.genome.jp/kegg/pathway.html, accessed in September 2019) [2, 3]. 5682 chemicals, encoded by KEGG IDs, and 792 enzymes, represented by EC numbers, were obtained. For the same representation of chemicals and enzymes in the constructed network, KEGG IDs of chemicals were map onto their PubChem IDs and specific human proteins of obtained EC numbers were extracted, which were further converted into Ensembl IDs. According to KEGG PATHWAY, these chemicals and enzymes were classified into 11 metabolic pathway types, which are listed in column 1 of Table 1. All above-obtained chemicals and enzymes were used as nodes in the constructed heterogeneous network that was described in “Heterogeneous Network Construction.” However, some nodes were isolated in the network and were discarded. As a result, we obtained 2329 chemicals (PubChem IDs) and 1124 human enzymes (Ensembl IDs). The number of chemicals and enzymes in each metabolic pathway type is listed in Table 1. Detailed chemicals and enzymes in each metabolic pathway type are provided in Table S1.

It is easy to see from Table 1 (last two rows) that the total number of chemicals/enzymes in eleven metabolic pathway types was larger than the number of different chemicals/enzymes. Thus, the problem of assigning chemicals and enzymes to metabolic pathway types was a multilabel classification problem. In this study, a uniform multilabel classifier was built to correctly predict metabolic pathway types of chemicals and enzymes.

### 2.2. Heterogeneous Network Construction

The chemicals and enzymes are the major components in metabolic pathways. The classic feature extraction methods always pick up essential features from the properties of themselves. With the development of the network technique, it provides another pipeline to access important features of chemicals and enzymes. Here, we adopted a network scheme to organize chemicals and enzymes.

To construct the network, we downloaded the information of CCIs and CPIs from STITCH (http://stitch.embl.de/, version 4.0) [17, 18]. Furthermore, the information of PPIs was retrieved from STRING (https://string-db.org/, version 10.0) [19, 20]. For CCIs, the file “chemical_chemical.links.v4.0.tsv.gz” was downloaded, from which we extracted the CCIs between 2329 chemicals. As a result, 82368 CCIs were obtained. Each CCI contained two chemicals, represented by PubChem IDs, and one confidence score with a range between 1 and 999. Such score integrated several types of associations derived from different aspects of chemicals, including structures, activities, reactions, and literature occurrence. Thus, such score can widely measure the associations of chemicals. For formulation, let us denote this score on the CCI between chemicals *c*_1_ and *c*_2_ as *S*_CCI_(*c*_1_, *c*_2_). For CPIs, we downloaded the file, named “9606.protein_chemical.links.v4.0.tsv.gz.” From this file, the CPIs between 2329 chemicals and 1124 enzymes were picked up, resulting in 41066 CPIs. Each CPI consists of one chemical and one protein, denoted by PubChem ID and Ensembl ID, respectively, and one confidence score with a range between 1 and 999. Such score is also obtained by evaluating several aspects of chemicals and proteins. The score between chemical *c* and protein *p* was denoted by *S*_CPI_(*c*, *p*). As for PPIs, the file “9606.protein.links.v10.txt.gz” in STRING was downloaded. We extracted PPIs between 1124 enzymes, obtaining 59868 PPIs. Two proteins, represented by Ensembl IDs, and one confidence score comprised each PPI. Likewise, the score integrated several types of associations of proteins and can widely measure their linkage, and its range is also between 1 and 999. For convenience, the score of proteins *p*_1_ and *p*_2_ was denoted by *S*_PPI_(*p*_1_, *p*_2_). Because all the above confidence score is between 1 and 999, we refined them by dividing it by 1000 so that the refined confidence score was between 0 and 1.

According to CCIs retrieved from STITCH, we constructed a chemical network. Such network defined 2329 chemicals as nodes. Two nodes were adjacent if and only if their corresponding chemicals can comprise a CCI with the refined confidence score larger than zero. Moreover, the refined confidence score was assigned to the corresponding edge as its weight. For convenience, such network was denoted by *N*_*C*_. A bipartite network was built according to CPIs retrieved from STITCH. Each edge connected a chemical node and an enzyme node if they can comprise a CPI with the refined confidence score higher than zero. Likewise, the refined confidence score was defined as the weight of the corresponding edge. This network was denoted as *N*_*C*−*P*_. The third protein network was constructed with the PPIs obtained from STRING. Two nodes were connected by an edge if and only if their corresponding proteins can comprise a PPI with the refined confidence score higher than zero. Also, the refined score was assigned to the edge as its weight. Such network was denoted by *N*_*P*_.

**Figure 1:**
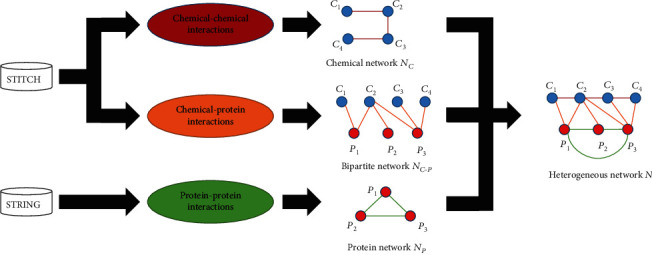
Procedures for constructing the heterogeneous network. From two public databases: STITCH and STRING, chemical-chemical, chemical-protein, and protein-protein interactions are downloaded. They are used to construct three networks, which are combined together to comprise the heterogeneous network.

The above-constructed three networks were combined to build a large heterogeneous network. For an easy description, this network was denoted as *N*. The construction procedures of *N* are illustrated in Figure 1.

### 2.3. Network Embedding Algorithm

A heterogeneous network *N* was built in the above section. Informative relationship between chemicals and enzymes was contained in such network. In this study, a powerful network embedding algorithm, Mashup [14], was employed to extract informative features of chemicals and enzymes. This algorithm has been adopted to deal with several biological and medical problems [12, 21–27]. Its brief description was as follows.

The Mashup consists of two stages to extract embedding features of nodes in a network, say *N*. In the first stage, each node *v*_*i*_ in *N* is picked up as the seed node of the random walk with restart (RWR) algorithm [28, 29]. When the RWR algorithm stops, probabilities assigned to all nodes are aligned together to comprise a raw feature vector of *v*_*i*_, denoted as *V*_*i*_. However, such vector has a high dimension. A dimensionality reduction procedure is necessary, which is done in the second stage. Let *X*_*i*_ be the final feature vector of *v*_*i*_ and *W*_*i*_ be the context feature vector of *v*_*i*_ in *N*. The purpose of the second stage is to determine the optimal components in these two vectors. Thus, an optimization problem is set up as follows:

(1) $$\begin{array}{c} \underset{X_{i},W_{i}}{\mathrm{minimize}}\;\frac{1}{n}\overset{n}{\underset{i=1}{\sum }}D_{\text{KL}}\left(V_{i}\left\|{\tilde{V}}_{i}\right.\right), \end{array}$$

where *n* stands for the number of nodes in network *N*, *D*_KL_(·) denotes the function of KL-divergence (relative entropy), the components in ${\tilde{V}}_{i}$ are defined as below

(2) $$\begin{array}{c} {\tilde{V}}_{i}^{k}=\frac{\exp\left({\left(X_{i}\right)}^{T}W_{k}\right)}{\sum_{k^{\prime }}\exp\left({\left(X_{i}\right)}^{T}W_{k^{\prime }}\right)}\;k=1,2,\cdots ,n. \end{array}$$

The outcome *X*_*i*_ was selected as the feature vector of *v*_*i*_, which would be used to construct the classification model.

This study adopted the Mashup program retrieved from http://cb.csail.mit.edu/cb/mashup/. For convenience, default parameters were used.

### 2.4. Multilabel Classifier (iMPTCE-Hnetwork)

Because some chemicals/enzymes can belong to two or more metabolic pathway types, the problem of assigning chemicals/enzymes to metabolic pathway types is evidently a multilabel classification problem. Generally, to deal with such problem, there are two types of schemes. The first one is problem transformation, that is, the original multilabel classification problem is transformed into several single-label classification problems. The second one is the algorithm adaption. This scheme extends the existing one single-label classification algorithm so that the new algorithm can process multilabel problems. In this study, we adopted the first scheme to build the classification model.

RAndom k-labELsets (RAKEL) [15] is a classic method to build multilabel classifiers, which can be deemed as the generalization of the Label Powerset (LP) algorithm. To date, this method has been applied to build several multilabel classifiers for tackling different biological and medical problems [9, 21, 30–34]. For a multilabel problem involving *m* labels (*m* = 11 in this study), let *L* = {*l*_1_, *l*_2_, ⋯, *l*_*m*_} be its label set. Select an integer *k* with 1 ≤ *k* ≤ *m* and construct all *k*-subsets of *L*. For one randomly selected *k*-subset *L*_*k*_, an LP classifier is constructed. In detail, members in the power set of *L*_*k*_ are defined as the new labels. And each sample is assigned a new label according to its original labels. For example, if a sample has two labels, say *l*_1_ and *l*_2_, a new label, representing {*l*_1_, *l*_2_}, is assigned to this sample. After that, each sample has only one new label, and a single-label classifier, called LP classifier, can be built based on a single-label classification algorithm. Clearly, multiple LP classifier with different *k*-subsets should be constructed because one *k*-subset cannot cover all labels. Thus, there are another parameters, *M*, of RAKEL to determine the number of LP classifiers. The final multilabel classifier integrates the above-constructed *m* LP classifiers.

Given a query sample, each LP classifier gives its binary decision on each label. The average of binary decisions on each label is computed. If the average is larger than a predefined threshold (generally, it is set to 0.5), the corresponding label is assigned to the sample.

In this study, we adopted the tool “RAKEL” in Meka (http://waikato.github.io/meka/) [35], which implements the RAKEL described above. As mentioned above, *k* and *M* are two major parameters of RAKEL. Several values were set to them for building an optimal multilabel classifier. For an easy description, the classifier constructed by RAKEL was called the RAKEL classifier.

### 2.5. Classification Algorithm

The RAKEL was used to construct a multi-label classifier. One basic single-label classification algorithm was necessary to set up multiple LP classifiers. Here, we selected one of the most classic classification algorithms, SVM [7], which has wide applications in bioinformatics [21, 30, 31, 36–40].

SVM is a statistical learning theory-based classification algorithm. The key point is to find out an optimal hyperplane, which can separate samples into two classes with maximum margin. However, in most cases, samples in their original space are not linearly separable, that is, such hyperplane cannot be found out. A kernel function is employed to map samples into another space with higher dimensions, in which samples are linearly separable. After the optimal hyperplane has been discovered, the class of a new sample is determined according to the side of the hyperplane it belongs to. The original SVM can only deal with binary problems. Some schemes (e.g., one-versus-others, one-versus-one) can be adopted so that it can tackle problems with multiple classes.

In this study, we selected the SVM whose training procedures are optimized by the sequential minimal optimization algorithm [41]. Such type of SVM is integrated in Meka. The tool “RAKEL” can directly invoke it. Two kernel functions (polynomial kernel, RBF kernel) were tried to select the best one.

### 2.6. Performance Evaluation

All constructed multilabel classifiers were evaluated by ten-fold cross-validation [42]. In such test, samples are divided into ten parts. Each part is singled out one by one as the test dataset, whereas the rest nine parts are used to training the classifier. As a result, each sample is tested exactly once.

According to the results of ten-fold cross-validation, some measurements can be computed. Here, we selected three measurements: accuracy, exact match, and hamming loss [9, 21, 30, 31]. Their definitions are as below

(3) $$\begin{array}{c} \left\{\begin{array}{c} \text{Accuracy}=\frac{1}{n}\overset{n}{\underset{i=1}{\sum }}\left(\frac{\left\|L_{i}\cap {L_{i}}^{\prime }\right\|}{\;|\;\left\|L_{i}\cup {L_{i}}^{\prime }\right\|}\right),\\ \text{Exact match}=\frac{1}{n}\overset{n}{\underset{i=1}{\sum }}\nabla \left(L_{i},{L_{i}}^{\prime }\right)\\ \text{Hamming loss}=\frac{1}{n}\overset{n}{\underset{i=1}{\sum }}\frac{\left\|L_{i}\Delta {L_{i}}^{\prime }\right\|}{m}, \end{array}\right., \end{array}$$

where *n* stands for the total number of samples, *m* represents the number of labels, *L*_*i*_ and *L*_*i*_′ denote the actual and predicted label set of the *i*-th sample, Δ indicates the symmetric difference operation, and ∇ is defined as follows:

(4) $$\begin{array}{c} \nabla \left(L_{i},{L_{i}}^{\prime }\right)=\left\{\begin{array}{cc} 1 & \text{if }L_{i}\text{ is identifical to }{L_{i}}^{\prime }\\ 0 & \text{otherwise} \end{array}.\right. \end{array}$$

Evidently, for accuracy and exact match, the higher they are, the better performance the classifier has. On the contrary, the lower the hamming loss is, the better the performance is. Furthermore, to give a uniform evaluation, we used the following equation to integrate the above three measurements

(5) $$\begin{array}{c} \text{Integrated score}=\text{Accurcay}\times \text{Exact match}\times \left(1‐\text{Hammong loss}\right). \end{array}$$

The classifier with a high integrated score indicated its high performance. We tried to construct a classifier with an integrated score as high as possible.

## 3. Results and Discussion

**Figure 2:**
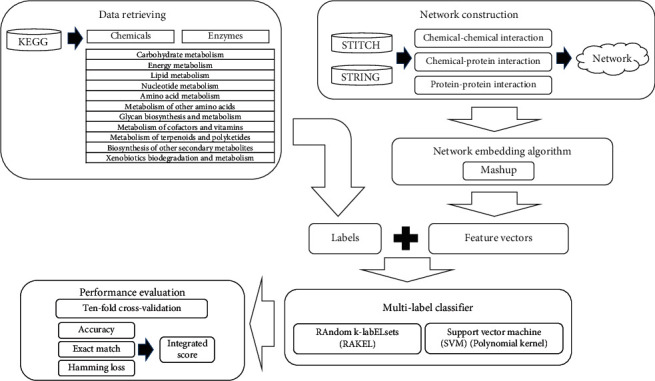
Entire procedures for constructing and evaluating the multilabel classifier to identify metabolic pathway types of chemicals and enzymes. The information of metabolic pathways is retrieved from KEGG, inducing the labels of chemicals and enzymes. Three types of interaction information are obtained from STITCH and STRING to construct a heterogeneous network. The network embedding algorithm, Mashup, is applied on the heterogeneous network to extract feature vectors of chemicals and enzymes. Labels and vectors are fed into the RAndom k-labELsets (RAKEL) algorithm, incorporating support vector machine (SVM) as the basic classifier, to construct the multilabel classifier. The classifier is evaluated by ten-fold cross-validation.

In this study, we proposed a multilabel classifier, called the iMPTCE-Hnetwork, to identify metabolic pathway types of chemicals and enzymes. The entire procedures are illustrated in Figure 2. This section gave evaluation results of such a classifier and elaborated its high utility.

### 3.1. Performance of the iMPTCE-Hnetwork

The constructed classifier used the feature vectors by applying Mashup on a heterogeneous network *N*. However, the dimension of the vector was a problem. Here, we tried six dimensions varying between 50 and 300 with an interval of 50. As for the main parameters *k* and *M* in RAKEL, *M* was set to 5 and 10, *k* was tried on each value between 2 and 11. Furthermore, SVM was selected as the basic classification algorithm. The kernel was set as the polynomial kernel, where the exponent (E) was set to 1, 2, and 3. Three values, including 1, 2, and 3, of regularization parameter *C* were tried. A lot of classifiers with all possible settings were constructed, which were further evaluated by ten-fold cross-validation.

The test results indicated that when *k* = 11, *M* = 5, *C* = 2, *E* = 1, and dimension = 250, the iMPTCE-Hnetwork yielded the highest integrated score, which was 0.591 (Table 2). The accuracy, exact match, and hamming loss were 0.818, 0.754, and 0.042 (Table 2), respectively. The accuracy was higher than 0.800, and the exact match exceeded 0.750, suggesting the good performance of the iMPTCE-Hnetwork.

**Figure 3:**
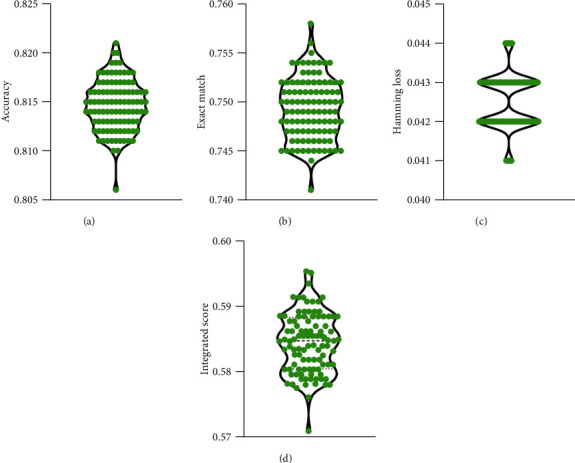
Violin plot to show the performance of the iMPTCE-Hnetwork under 100 ten-fold cross-validation. (a) Accuracy; (b) exact match; (c) hamming loss; (d) integrated score.

Besides, to fully evaluate the performance of iMPTCE-Hnetwork with ten-fold cross-validation, we further did the ten-fold cross-validation 100 times. The obtained values of accuracy, exact match, hamming loss, and integrated score induced four violin plots, as shown in Figure 3. It can be observed that accuracy varied between 0.805 and 0.825, the exact match changed between 0.740 and 0.760, the hamming loss was between 0.040 and 0.045, and the integrated score varied between 0.570 and 0.600. These results implied that the iMPTCE-Hnetwork was quite stable for different divisions of samples.

### 3.2. Analysis of the Effects of Heterogeneous Network

The proposed classifier, iMPTCE-Hnetwork, adopted the chemical and enzyme features derived from the heterogeneous network *N*. Clearly, the accuracy of *N* is an essential factor which can influence the performance of the classifier. In this section, an analysis would be given to indicate the importance of the heterogeneous network. Furthermore, we also analyzed the contributions of chemical and protein networks for building the classifier.

**Figure 4:**
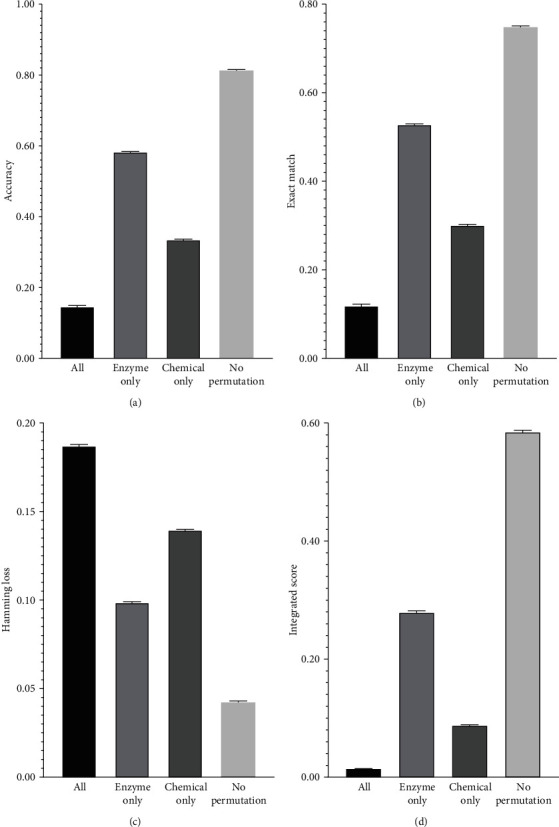
Bar chart to illustrate the performance of the RAKEL classifiers with SVM as the basic classification algorithm under the permutation of nodes in the heterogeneous network. “All” indicates that both chemical and protein nodes are permutated; “Enzyme only” indicates that only protein nodes are permutated; “Chemical only” indicates that only chemical nodes are permutated; “No permutation” indicates no permutation is done for chemical and protein nodes (i.e., the proposed classifier, iMPTCE-Hnetwork). (a) Accuracy; (b) exact match; (c) hamming loss; (d) integrated score.

For the heterogeneous network *N*, a permutation was done for nodes (representing enzymes) and nodes (denoting chemicals), respectively. Obtained features (250-D) were fed into the RAKEL to construct the classifier. The same parameter setting (*k* = 11, *M* = 5, *C* = 2, *E* = 1) was used. Such a classifier was assessed by ten-fold cross-validation. To make the results more reliable, the above procedures were done 100 times, resulting in 100 values of accuracy, exact match, hamming loss, and integrated score. These values are shown in Figure 4, in which the performance of iMPTCE-Hnetwork is also listed. It can be observed that the permutation made the performance of the classifier very poor. Compared with the performance of iMPTCE-Hnetwork, the accuracy, exact match, and integrated score reduced about 0.667, 0.630, and 0.570, respectively, whereas hamming loss increased about 0.145. It is indicated that the accuracy of heterogeneous network *N* is very important to construct the efficient classifier.

In addition, we also analyzed the importance of chemical and protein networks for constructing the classifier. First, we only permutated the protein (enzyme) nodes. Features (250-D) yielded by Mashup were used to construct the RAKEL classifier (*k* = 11, *M* = 5, *C* = 2, *E* = 1), which was also evaluated by ten-fold cross-validation. Such procedures were also done 100 times. The average performance is shown in Figure 4. Evidently, the classifier became worse. The accuracy, exact match, and integrated score declined about 0.230, 0.220, and 0.306, respectively, whereas hamming loss increased about 0.056. Second, the above procedures were done for chemical nodes. Results are illustrated in Figure 4. Also, the performance of the classifier decreased. In detail, accuracy, exact match, and integrated score were about 0.478, 0.448, and 0.497, respectively, lower than those of the iMPTCE-Hnetwork, and the hamming loss was about 0.097 higher than that of the iMPTCE-Hnetwork. It can be observed that when chemical nodes were permutated, the performance of the classifier was much lower than that of the classifier with enzyme permutation, suggesting that the chemical network gave more contribution to build the classifier.

### 3.3. The Superiority of Combining the Information of Chemicals and Enzymes

As mentioned above, iMPTCE-Hnetwork provided good performance for the identification of metabolic pathway types of chemicals and enzymes. From its construction procedures, we can see that the information of chemicals and enzymes was poured into a uniform system, that is, the information of chemicals can be used to predict metabolic pathway types of enzymes and vice versa. This fact may lead to the superiority of the classifier. Here, we gave some analyses.

There were two essential stages that the information of chemicals and enzymes was utilized with each other. The first stage was the feature extraction. Because chemicals and enzymes have several different points, an ordinary method may only consider chemicals (enzymes) when extracting chemical (enzyme) features and excluding the information of enzymes (chemicals). In our classifier, chemicals and enzymes were all deemed as nodes in the heterogeneous network *N*, that is, they were combined together to extract features. The second essential stage was the classification. In the iMPTCE-Hnetwork, the enzyme features were used to predict the metabolic pathway types of chemicals and vice versa. Ordinary methods may separate the classification procedures, i.e., the prediction of metabolic pathway types of chemicals (enzymes) only used the chemical (enzyme) features. Considering the above two stages, we did the following two tests.

**Figure 5:**
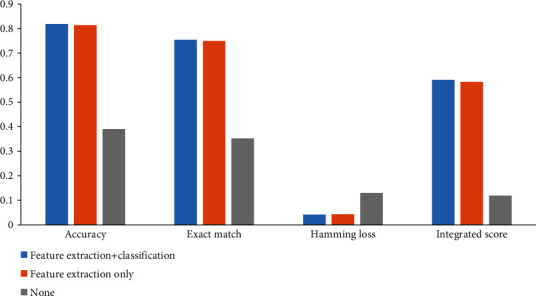
Bar chart to illustrate the performance of classifiers using different combination stages of chemical and enzyme information. When the combination of chemical and enzyme information is used in both feature extraction and classification procedures, the classifier provides the best performance.

For the first test, we extracted chemical features from the chemical network *N*_*C*_ and enzyme features from the protein network *N*_*P*_ with Mashup. In this case, the feature extraction procedures separated the information of chemicals and enzymes. Because we did not know which dimension was best, six dimensions from 50 to 300 with an interval of 50 were obtained. With a given dimension, a multilabel classifier was set up for chemicals and enzyme, respectively. We used the same parameter setting of iMPTCE-Hnetwork. Each classifier was evaluated by ten-fold cross-validation. For each combination of dimensions of chemicals and enzymes, we combined the cross-validation results to compute four measurements mentioned in “Performance Evaluation.” As a result, when the dimensions of chemicals and enzymes were all 100, we obtained the highest integrated score (0.120). The accuracy was 0.390, the exact match was 0.352, and hamming loss was 0.130, which are shown in Figure 5. It can be observed that such performance was much lower than that of the iMPTCE-Hnetwork, implying feature extraction with the combination of chemicals and enzymes improved the quality of chemical and enzyme features.

The second test was for the classification procedure. We used the 250-D feature vectors of the iMPTCE-Hnetwork. However, the classification procedures strictly separated chemicals and enzymes. The predicted results of chemicals and enzymes were combined to calculate four measurements. The integrated score was 0.583, the accuracy was 0.814, the exact match was 0.749, and the hamming loss was 0.043, as shown in Figure 5. This performance was slightly lower than that of the iMPTCE-Hnetwork, implying the classification procedure by combining chemical and enzyme features can also improve the performance of the classifier. However, its influence was much smaller than that of the feature extraction.

With the above arguments, the combination of chemicals and enzymes is an important aspect to cause the good performance of our classifier.

### 3.4. Comparison of the RAKEL Classifiers with Different Classification Algorithms

The classifier, iMPTCE-Hnetwork, was built based on SVM (polynomial kernel). In fact, we also tried SVM (RBF kernel) and random forest (RF) [43]. Like SVM, RF is also a widely used and powerful classification algorithm [8, 11, 22, 44–47]. For SVM (RBF kernel), the same values of regularization parameter *C* were tried, and *γ* was set to 0.01, 0.02, and 0.03. As for RF, the main parameter, the number of decision trees, was set to different values from 10 to 100 with an interval of 10. Same dimensions and parameters of RAKEL (*M* and *k*), which were tried when constructing iMPTCE-Hnetwork, were also used. Each classifier was also assessed by ten-fold cross-validation. The best performance for SVM (RBF kernel) and RF is listed in Table 2. Four measurements for SVM (RBF kernel) were 0.757, 0.670, 0.055, and 0.479, respectively, whereas they were 0.803, 0.743, 0.045, and 0.570, respectively, when the basic classifier was RF. Compared with the performance of iMPTCE-Hnetwork, also listed in Table 2, their performance was less or more lower. For the integrated score, they were about 0.112 and 0.021 lower, respectively. The RAKEL classifier with RF was slightly inferior to iMPTCE-Hnetwork, but the performance of RAKEL classifier with SVM (RBF kernel) was much lower than that of the iMPTCE-Hnetwork. It is suggested that the selection of SVM (polynomial kernel) as the basic classifier was a relatively proper choice.

### 3.5. Comparison of the BR Classifiers

The RAKEL algorithm is an efficient scheme for tackling multilabel classification problems. The Binary Relevance (BR) [16] method is another widely used scheme. This scheme adopted the one-against-all strategy to construct several binary classifiers for each label and integrated them together. In fact, if the parameter *k* of RAKEL is set to 1, RAKEL is the same as BR. Here, we compared the classifiers based on BR with RAKEL classifiers. For convenience, the classifier based on BR was called the BR classifier. Three basic classifiers: SVM (polynomial kernel), SVM (RBF kernel), and RF, were adopted to construct the BR classifiers. All parameter settings mentioned above were tried. Each BR classifier was evaluated by ten-fold cross-validation. The best performance for each basic classifier is listed in Table 2.

The BR classifier with SVM (polynomial kernel) yielded the accuracy of 0.786, the exact match of 0.690, the hamming loss of 0.043, and the integrated score of 0.519. When the basic classifier was SVM (RBF kernel), the BR classifier provided the accuracy of 0.598, the exact match of 0.533, the hamming loss of 0.058, and the integrated score of 0.300. As for the RF, its BR classifier generated the accuracy of 0.666, the exact match of 0.602, the hamming loss of 0.052, and the integrated score of 0.380. Each measurement was lower than the corresponding one of the iMPTCE-Hnetwork, suggesting that RAKEL was more efficient than BR for the identification of the metabolic pathway types of chemicals and enzymes. Furthermore, three basic classifiers gave the same strength for building the BR classifiers as for building the RAKEL classifiers, that is, the SVM (polynomial kernel) was the best, followed by RF and SVM (RBF kernel).

### 3.6. Comparison of Classifiers with Other Embedding Features

The iMPTCE-Hnetwork was constructed using the chemical and enzyme features derived from the heterogeneous network by Mashup. To date, several network embedding algorithms have been proposed and applied to deal with some realistic problems. Here, we selected two of them to make comparisons. They were DeepWalk [48] and Node2vec [49]. These two algorithms adopted quite different schemes to extract features for representing nodes. They always produce a lot of paths for each node. Each path is deemed as a sentence, and nodes in the paths are termed as words. Then, Word2vec [50] is applied to these sentences to extract features. The main difference of these two algorithms is the way to produce paths. Node2vec adopts a more advanced scheme and thus is considered to be more powerful than DeepWalk. We downloaded the DeepWalk program at https://github.com/phanein/deepwalk, and the program of Node2vec was retrieved from https://snap.stanford.edu/node2vec/. They were all applied to the heterogeneous network *N* with their default parameters. Likewise, the dimension was set to 50 to 300 with an interval of 50.

Features yielded by DeepWalk and Node2vec were fed into RAKEL with different values of *M* and *k* and different basic classifiers (SVM (polynomial kernel), SVM (RBF kernel), and RF) to construct RAKEL classifiers. All classifiers were evaluated by ten-fold cross-validation. For features yielded by DeepWalk, the best performance of RAKEL classifiers with one of the three basic classifiers is shown in Table 3. It can be seen that these classifiers all gave a poor performance. The accuracy was all lower than 0.350, the exact match was lower than 0.310, the hamming loss was higher than 0.140, and the integrated score was lower than 0.090. Compared with the measurements of RAKEL classifiers using features produced by Mashup (Table 2), they were much lower. As for the features generated by Node2vec, RAKEL classifiers gave better performance. Four measurements are listed in Table 4. The accuracy was higher than 0.700, the exact match was higher than 0.650, the hamming loss was lower than 0.060, and the integrated score was higher than 0.460. However, they were still inferior to those of the RAKEL classifiers using features produced by Mashup (Table 2). It can be concluded that the features yielded by the Mashup were more informative than those produced by DeepWalk and Node2vec for the identification of metabolic pathway types of chemicals and enzymes.

Besides, to fully elaborate the conclusion in the above paragraph, we also used the features yielded by DeepWalk and Node2vec to build BR classifiers. The ten-fold cross-validation results are listed in Tables 3 and 4, respectively. For BR classifiers using features yielded by DeepWalk, their performance was still very poor. Given the same basic classifier, such BR classifier was much inferior to that with features yielded by Mashup. The BR classifier with features yielded by Node2vec gave much better performance. When the basic classifier was SVM (RBF kernel), the BR classifier was slightly superior to the BR classifier using features yielded by Mashup. However, the other two basic classifiers still generated lower performance. In addition, the best BR classifier using features yielded by Mashup was much better than the best BR classifier using features yielded by Node2vec. These results further confirmed that the features yielded by the Mashup were more efficient, which was an important reason why iMPTCE-Hnetwork can provide such high performance.

## 4. Conclusions

This study proposed an efficient multilabel classifier for the identification of metabolic pathway types of chemicals and enzymes. We did several tests to elaborate its rationality, including parameter settings, selection of basic classifiers, scheme for tackling multilabel problems, and network embedding algorithms. The main merit of the proposed classifier is the integration of chemical and enzyme information. Their information is utilized with each other in both feature extraction and classification procedures. It is hopeful that this classifier can be a useful tool for investigating the metabolic pathway system.

## Figures and Tables

**Table 1:** Number of chemicals and enzymes in each metabolic pathway type.

Metabolic pathway type	Number of chemicals	Number of enzymes
Carbohydrate metabolism	399	274
Energy metabolism	153	102
Lipid metabolism	405	337
Nucleotide metabolism	135	113
Amino acid metabolism	427	215
Metabolism of other amino acids	132	106
Glycan biosynthesis and metabolism	22	156
Metabolism of cofactors and vitamins	303	175
Metabolism of terpenoids and polyketides	195	46
Biosynthesis of other secondary metabolites	326	54
Xenobiotics biodegradation and metabolism	422	141
Total numbers of chemicals/enzymes in all pathway types	2919	1719
Number of different chemicals/enzymes	2329	1124

**Table 2:** Performance of different multilabel classifiers based on features yielded by Mashup under ten-fold cross-validation.

Scheme	Basic classification algorithm	Accuracy	Exact match	Hamming loss	Integrated score
RAKEL	Support vector machine (polynomial kernel)	0.818	0.754	0.042	0.591
	Support vector machine (RBF kernel)	0.757	0.670	0.055	0.479
	Random forest	0.803	0.743	0.045	0.570
Binary relevance	Support vector machine (polynomial kernel)	0.786	0.690	0.043	0.519
	Support vector machine (RBF kernel)	0.598	0.533	0.058	0.300
	Random forest	0.666	0.602	0.052	0.380

**Table 3:** Performance of different multilabel classifiers based on features yielded by DeepWalk under ten-fold cross-validation.

Scheme	Basic classification algorithm	Accuracy	Exact match	Hamming loss	Integrated score
RAKEL	Support vector machine (polynomial kernel)	0.333	0.290	0.145	0.083
	Support vector machine (RBF kernel)	0.342	0.304	0.140	0.089
	Random forest	0.334	0.297	0.142	0.085
Binary relevance	Support vector machine (polynomial kernel)	0.297	0.213	0.143	0.054
	Support vector machine (RBF kernel)	0.059	0.057	0.118	0.003
	Random forest	0.142	0.118	0.121	0.015

**Table 4:** Performance of different multilabel classifiers based on features yielded by Node2vec under ten-fold cross-validation.

Scheme	Basic classification algorithm	Accuracy	Exact match	Hamming loss	Integrated score
RAKEL	Support vector machine (polynomial kernel)	0.774	0.698	0.050	0.513
	Support vector machine (RBF kernel)	0.738	0.668	0.059	0.464
	Random forest	0.762	0.695	0.054	0.501
Binary relevance	Support vector machine (polynomial kernel)	0.734	0.631	0.049	0.440
	Support vector machine (RBF kernel)	0.652	0.574	0.058	0.353
	Random forest	0.639	0.581	0.058	0.350

## Data Availability

The original data used to support the findings of this study are available at the KEGG PATHWAY and in the supplementary information files.

## Supplementary Materials

Supplementary Materials Table S1: Chemicals and enzymes in each metabolic pathway.
