# Supplementary material for: iMPTCE-Hnetwork: A Multilabel Classifier for Identifying Metabolic Pathway Types of Chemicals and Enzymes with a Heterogeneous Network
Source: Comput Math Methods Med. 2021 Jan 4;2021:6683051. doi: 10.1155/2021/6683051 (PMC7803417; doi:10.1155/2021/6683051)
Supplement: Supplementary Materials — Table S1: Chemicals and enzymes in each metabolic pathway. [file 6683051.f1.pdf]

Table S1. Chemicals and enzymes in each metabolic pathway type.

1. Carbohydrate metabolism

CID000001060	CID000439183	CID000440641	CID000439184	CID000008629
CID000444493	CID000064689	CID000010267	CID000161227	CID000000753
CID000005793	CID000439191	CID000440936	CID000439190	CID000017473
CID000000175	CID000066370	CID000440938	CID000009290	CID000135191
CID000000970	CID000000702	CID000000051	CID000439194	CID000094715
CID000001132	CID000439278	CID000001110	CID000122357	CID000439195
CID000001005	CID000439284	CID000092133	CID000091493	CID000000756
CID000000177	CID000186004	CID000444972	CID000079014	CID000439203
CID000065533	CID000439427	CID000222656	CID000439236	CID000439204
CID000000668	CID000439503	CID000000311	CID005460448	CID000439205
CID000439168	CID000440568	CID000001198	CID000439288	CID000439215
CID000107689	CID000440969	CID000643757	CID005460191	CID000006912
CID000006251	CID011020241	CID000000972	CID000439452	CID000000523
CID000439231	CID005459849	CID000440649	CID000073563	CID024892720
CID000000870	CID000439290	CID000439167	CID000000046	CID000439240
CID000025310	CID000192781	CID000007339	CID000165007	CID005460056
CID000439279	CID000033037	CID000005779	CID000193886	CID000439245
CID000000618	CID003037582	CID000007027	CID003035456	CID003246006
CID000105021	CID000439373	CID023724466	CID017756769	CID000439255
CID000005780	CID005483640	CID011006912	CID017756767	CID000439264
CID000447152	CID000191190	CID000449043	CID044229075	CID000439273
CID000017106	CID006857365	CID000439536	CID024798720	CID000165130
CID000439394	CID000001122	CID000439544	CID025203873	CID000000604
CID000152306	CID005460407	CID000439572	CID046174047	CID000439315
CID000439446	CID000439583	CID023724469	CID024755535	CID000439343
CID022833608	CID000439695	CID000439709	CID024755534	CID000151104
CID000439507	CID000092283	CID005462244	CID056927739	CID000439399
CID000439545	CID000440667	CID000440001	CID054588960	CID000439508
CID006857362	CID010130220	CID014427002	CID045281255	CID000094154
CID005459988	CID023724606	CID024892738	CID000000868	CID000439692
CID005460171	CID023724607	CID023724475	CID000439294	CID000439694
CID000065369	CID000005958	CID011426583	CID000107737	CID000439723
CID000439912	CID000439178	CID000193002	CID000000890	CID023615403
CID000193537	CID000439186	CID000440364	CID000439444	CID005460049
CID006857359	CID000439193	CID016069659	CID000439455	CID000440303
CID000440940	CID046173703	CID000188960	CID000439456	CID000440390
CID000443242	CID000016500	CID000440961	CID000107758	CID000439432
CID023724458	CID000439244	CID000440962	CID000439468	CID006971042
CID000439160	CID000122250	CID000443243	CID000440043	CID000644110
CID000005988	CID000122336	CID000439350	CID000440194	CID000440470
CID000006036	CID046173710	CID000439459	CID000128419	CID000439163
CID000000892	CID000007427	CID000006318	CID000440211	CID000018396
CID000062223	CID000082400	CID054675756	CID024771766	CID000018950
CID000087916	CID000439555	CID005280523	CID000121969	CID000439192

CID000439242	CID000439762	CID000000254	CID000121920	CID000065127
CID000439312	CID000439874	CID000000371	CID000440388	CID010918995
CID000128869	CID000440376	CID000440018	CID011706915	CID000016323
CID000439357	CID000440773	CID005280595	CID000441201	CID000007095
CID000035717	CID000440946	CID000000569	CID000441202	CID000015625
CID011727586	CID000644101	CID000003035	CID000443266	CID000000568
CID000439531	CID000167489	CID000003036	CID012308755	CID000009216
CID000011850	CID000445675	CID000440867	CID000000283	CID000006854
CID000024154	CID000448209	CID000039929	CID000644066	CID000036187
CID000439781	CID000439174	CID000042128	CID000000289	CID000063090
CID000097165	CID023724459	CID000051130	CID000000227	CID000038439
CID000440098	CID023724461	CID000038199	CID000000242	CID000038251
CID000440117	CID000439197	CID000051043	CID000000186	CID000042540
CID000440654	CID000439213	CID000036231	CID000061503	CID000029575
CID016048618	CID000440997	CID000036188	CID000092153	CID000033032
CID000440658	CID000440996	CID000053036	CID000000880	CID000000784
CID000439620	CID000439281	CID000035823	CID005280361	CID000000750
CID000439659	CID003086191	CID000047650	CID000000338	CID000000760
CID000168934	CID000000058	CID000038019	CID000000261	CID000005961
CID000439945	CID000000887	CID000050891	CID000447765	CID000005951
CID000000524	CID000001032	CID000040479	CID011966119	CID000092753
CID011966146	CID000000096	CID000018636	CID009543049	CID000122283
CID000440064	CID000000180	CID000000977	CID000000266	CID000000757
CID011966145	CID000000527	CID000000280	CID000000043	CID000000964
CID071448895	CID000001030	CID000000222	CID000225936	CID000000971
CID011966166	CID000010110	CID000439681	CID011966206	CID011966123
CID049852422	CID003081970	CID003080625	CID000000263	CID000201434
CID071448969	CID009543026	CID005459784	CID011966192	CID011966111
CID000000239	CID000068152	CID000008299	CID000000811	CID003082032
CID000445127	CID000003776	CID011966171	CID011966125	CID005497143
CID000092824	CID000000487	CID000001031	CID000643798	CID011966122
CID000000650	CID000000119	CID000440863	CID000444212	CID000119400
CID000439314	CID000123658	CID025137843	CID000439766	CID054678503
CID000010413	CID000449534	CID005462303	CID000441696	CID000000529
CID000092135	CID000000174	CID000006584	CID000444266	CID000000599
CID009543037	CID000638129	CID000001112	CID000000264	
ENSP00000260386	ENSP00000303246	ENSP00000288078	ENSP00000263035	ENSP00000209668
ENSP00000272117	ENSP00000217455	ENSP00000309092	ENSP00000222673	ENSP00000378359
ENSP00000263370	ENSP00000380514	ENSP00000179259	ENSP00000264932	ENSP00000296412
ENSP00000267615	ENSP00000238618	ENSP00000370194	ENSP00000364649	ENSP00000312606
ENSP00000262039	ENSP00000378161	ENSP00000216259	ENSP00000335304	ENSP00000280706
ENSP00000363046	ENSP00000372112	ENSP00000268261	ENSP00000342056	ENSP00000302393
ENSP00000365757	ENSP00000362463	ENSP00000369055	ENSP00000253792	ENSP00000229319
ENSP00000347032	ENSP00000307900	ENSP00000225614	ENSP00000355518	ENSP00000280704
ENSP00000264380	ENSP00000318868	ENSP00000368119	ENSP00000309477	ENSP00000222286
ENSP00000263967	ENSP00000333667	ENSP00000264382	ENSP00000216254	ENSP00000366927
ENSP00000289153	ENSP00000389175	ENSP00000326227	ENSP00000377446	ENSP00000345774
ENSP00000366563	ENSP00000244217	ENSP00000305692	ENSP00000366620	ENSP00000255084

ENSP00000352121	ENSP00000274813	ENSP00000218516	ENSP00000270776	ENSP00000387123
ENSP00000265970	ENSP00000365462	ENSP00000306920	ENSP00000377192	ENSP00000346827
ENSP00000356155	ENSP00000359680	ENSP00000216075	ENSP00000358931	ENSP00000261733
ENSP00000287996	ENSP00000241052	ENSP00000358727	ENSP00000280605	ENSP00000225740
ENSP00000359665	ENSP00000368066	ENSP00000345023	ENSP00000321259	ENSP00000295266
ENSP00000264864	ENSP00000316339	ENSP00000370718	ENSP00000306817	ENSP00000307241
ENSP00000357883	ENSP00000293217	ENSP00000350265	ENSP00000361512	ENSP00000205402
ENSP00000265382	ENSP00000348775	ENSP00000369456	ENSP00000381504	ENSP00000280346
ENSP00000335333	ENSP00000242592	ENSP00000258662	ENSP00000416583	ENSP00000346643
ENSP00000373399	ENSP00000231887	ENSP00000216962	ENSP00000283646	ENSP00000290573
ENSP00000219789	ENSP00000370023	ENSP00000216392	ENSP00000303356	ENSP00000292432
ENSP00000362115	ENSP00000357535	ENSP00000164139	ENSP00000267814	ENSP00000223366
ENSP00000360777	ENSP00000268251	ENSP00000317904	ENSP00000221403	ENSP00000269848
ENSP00000360154	ENSP00000269980	ENSP00000261195	ENSP00000285930	ENSP00000370517
ENSP00000352575	ENSP00000318351	ENSP00000410833	ENSP00000319501	ENSP00000312250
ENSP00000347792	ENSP00000359151	ENSP00000298198	ENSP00000298248	ENSP00000320171
ENSP00000357583	ENSP00000352706	ENSP00000330484	ENSP00000343838	ENSP00000339933
ENSP00000384534	ENSP00000262430	ENSP00000359096	ENSP00000304845	ENSP00000362413
ENSP00000359423	ENSP00000289416	ENSP00000359100	ENSP00000418532	ENSP00000305995
ENSP00000359417	ENSP00000296424	ENSP00000264029	ENSP00000362508	ENSP00000364475
ENSP00000345752	ENSP00000168216	ENSP00000420449	ENSP00000362513	ENSP00000364486
ENSP00000384651	ENSP00000322706	ENSP00000354347	ENSP00000303174	ENSP00000361064
ENSP00000371221	ENSP00000358414	ENSP00000253778	ENSP00000362525	ENSP00000364512
ENSP00000180173	ENSP00000196371	ENSP00000268097	ENSP00000362549	ENSP00000269097
ENSP00000074304	ENSP00000361914	ENSP00000261416	ENSP00000346768	ENSP00000253801
ENSP00000262992	ENSP00000381654	ENSP00000337854	ENSP00000251566	ENSP00000319814
ENSP00000361021	ENSP00000363614	ENSP00000356218	ENSP00000305221	ENSP00000216780
ENSP00000298229	ENSP00000314649	ENSP00000299498	ENSP00000304811	ENSP00000226253
ENSP00000337972	ENSP00000350928	ENSP00000358695	ENSP00000274278	ENSP00000345555
ENSP00000338185	ENSP00000259271	ENSP00000216410	ENSP00000282507	ENSP00000234590
ENSP00000260402	ENSP00000324842	ENSP00000210444	ENSP00000265403	ENSP00000324105
ENSP00000279230	ENSP00000267436	ENSP00000379839	ENSP00000341045	ENSP00000229277
ENSP00000388631	ENSP00000219054	ENSP00000356903	ENSP00000320401	ENSP00000272252
ENSP00000244007	ENSP00000327453	ENSP00000229329	ENSP00000334276	ENSP00000359991
ENSP00000345988	ENSP00000300441	ENSP00000302441	ENSP00000207870	ENSP00000297283
ENSP00000367747	ENSP00000301956	ENSP00000341828	ENSP00000385478	ENSP00000412189
ENSP00000266505	ENSP00000382349	ENSP00000311876	ENSP00000338703	ENSP00000342032
ENSP00000356015	ENSP00000327916	ENSP00000295448	ENSP00000302728	ENSP00000371393
ENSP00000265838	ENSP00000340296	ENSP00000387019	ENSP00000369442	ENSP00000316924
ENSP00000285093	ENSP00000366682	ENSP00000377303	ENSP00000364145	ENSP00000327070
ENSP00000321070	ENSP00000364883	ENSP00000262644	ENSP00000356047	ENSP00000299518
ENSP00000358719	ENSP00000331897	ENSP00000408526	ENSP00000369100	ENSP00000260985
ENSP00000313432	ENSP00000300051	ENSP00000269159	ENSP00000232375	

2. Energy metabolism

CID000000961	CID000000674	CID000439237	CID000006329	CID000000311
CID000005957	CID000001146	CID049859699	CID000000186	CID000000868
CID000005892	CID000160226	CID011966111	CID000439190	CID000001112
CID000439153	CID000439278	CID005497143	CID000000281	CID000122347

CID000000977	CID021122601	CID009543026	CID000439194	CID000000767
CID000006022	CID000123996	CID000010413	CID000000071	CID000001198
CID000001003	CID000068841	CID000068152	CID000172312	CID000092153
CID000001023	CID000189122	CID009543037	CID000005610	CID000643757
CID000001110	CID000439377	CID000449534	CID000000491	CID009825434
CID000643976	CID000448348	CID011966171	CID000026259	CID000439175
CID000000783	CID000001145	CID071448895	CID000023736	CID000222656
CID000444972	CID005462234	CID011966166	CID000000563	CID000000964
CID005280344	CID005462235	CID011966192	CID000006213	CID000000670
CID005280346	CID000000297	CID071448968	CID000000679	CID000439183
CID000000983	CID000000105	CID000000222	CID000006395	CID000439184
CID000005884	CID000000598	CID000033032	CID000123658	CID000010238
CID000005885	CID000000893	CID000005961	CID000439645	CID000001123
CID000023930	CID011966145	CID000024529	CID000165007	CID000001084
CID010219885	CID000440314	CID000000278	CID000000051	CID000000878
CID024892729	CID053297335	CID000000787	CID000644066	CID000001068
CID000000280	CID005460331	CID000000943	CID000092133	CID000099478
CID000001060	CID000440717	CID000000713	CID000092753	CID000439406
CID000444493	CID000193886	CID000145068	CID000006057	CID000122357
CID000000175	CID000443250	CID000000947	CID000439160	CID000164735
CID000000970	CID000447123	CID000000948	CID000091443	CID000000177
CID000000750	CID000086962	CID000000540	CID000000668	CID000000402
CID000000760	CID000146903	CID000000276	CID000439168	CID000001099
CID000000283	CID000169732	CID000009321	CID000000887	CID000005862
CID000005951	CID045266867	CID000010214	CID000005960	CID000091552
CID000000712	CID045480622	CID000159296	CID000439167	CID000439191
CID000001005	CID000005950	CID000001117		
ENSP00000264932	ENSP00000364486	ENSP00000222286	ENSP00000168216	ENSP00000285379
ENSP00000364649	ENSP00000226253	ENSP00000362413	ENSP00000356015	ENSP00000285381
ENSP00000356972	ENSP00000345555	ENSP00000305995	ENSP00000265838	ENSP00000300900
ENSP00000263774	ENSP00000234590	ENSP00000358931	ENSP00000285093	ENSP00000314099
ENSP00000233627	ENSP00000324105	ENSP00000280605	ENSP00000370023	ENSP00000366662
ENSP00000315774	ENSP00000229277	ENSP00000283646	ENSP00000357535	ENSP00000345659
ENSP00000322450	ENSP00000359991	ENSP00000303077	ENSP00000244217	ENSP00000367608
ENSP00000327268	ENSP00000297283	ENSP00000359539	ENSP00000274813	ENSP00000265174
ENSP00000354499	ENSP00000412189	ENSP00000245206	ENSP00000365462	ENSP00000406157
ENSP00000343885	ENSP00000342032	ENSP00000378408	ENSP00000365775	ENSP00000292147
ENSP00000362329	ENSP00000316924	ENSP00000345282	ENSP00000379108	ENSP00000266971
ENSP00000357835	ENSP00000327070	ENSP00000358719	ENSP00000377617	ENSP00000357861
ENSP00000271620	ENSP00000318868	ENSP00000260985	ENSP00000356290	ENSP00000249042
ENSP00000354687	ENSP00000333667	ENSP00000331897	ENSP00000277865	ENSP00000318852
ENSP00000355046	ENSP00000365773	ENSP00000253792	ENSP00000327589	ENSP00000262644
ENSP00000355206	ENSP00000215904	ENSP00000355518	ENSP00000307900	ENSP00000306397
ENSP00000354728	ENSP00000275605	ENSP00000309477	ENSP00000402608	ENSP00000262030
ENSP00000354961	ENSP00000296412	ENSP00000216254	ENSP00000178638	ENSP00000273398
ENSP00000354813	ENSP00000356729	ENSP00000377446	ENSP00000318912	ENSP00000262623
ENSP00000354665	ENSP00000367992	ENSP00000231887	ENSP00000358107	ENSP00000269848
ENSP00000370517	ENSP00000364475			

3. Lipid metabolism

CID000444493	CID006449797	CID000164673	CID000105074	CID005280914
CID000644066	CID000101770	CID000127601	CID000065517	CID005280915
CID000644109	CID000440371	CID005283827	CID000008629	CID005282263
CID000000985	CID023724484	CID005283886	CID000439162	CID005311211
CID000000867	CID000440558	CID005283882	CID000065533	CID005283159
CID000445639	CID000440559	CID005283853	CID000000668	CID005283157
CID000005281	CID000440560	CID005283851	CID000000753	CID005283202
CID000002969	CID000092746	CID000160636	CID000000670	CID005283203
CID000003893	CID000440670	CID000006128	CID000439183	CID005283204
CID000092753	CID005280793	CID000005994	CID000439194	CID005283205
CID000000379	CID005280794	CID000005870	CID000079014	CID005283146
CID000011005	CID005280795	CID000005879	CID000439278	CID005283147
CID000445638	CID005281327	CID000006013	CID000075049	CID005283154
CID000122283	CID005281326	CID000222865	CID000010442	CID005283155
CID000165436	CID000443212	CID000005754	CID016048618	CID009548884
CID000445344	CID000443237	CID000222786	CID000443243	CID005283168
CID011966124	CID000193524	CID000005757	CID000000177	CID011954042
CID011966110	CID023724571	CID000011222	CID000000305	CID011954043
CID011966179	CID010894570	CID000006238	CID000000700	CID005283162
CID000169621	CID053297366	CID000005881	CID000013804	CID005283211
CID011966177	CID023724575	CID000005839	CID000001015	CID005283208
CID011966197	CID023724596	CID000008955	CID000439249	CID005283209
CID011966173	CID023724597	CID000005753	CID000123727	CID005283210
CID011966169	CID023724604	CID000000129	CID000001014	CID009548885
CID011966158	CID000000750	CID000068570	CID000439276	CID011954059
CID011966155	CID000001123	CID003001028	CID000439285	CID009548886
CID011966216	CID000221493	CID000006166	CID000439436	CID005283156
CID011966162	CID000439479	CID000092810	CID000444183	CID009548880
CID011966160	CID071448889	CID000440114	CID000000187	CID005283163
CID003082152	CID000010140	CID000440143	CID000007618	CID005280450
CID000449118	CID000010133	CID000010635	CID000008113	CID005280581
CID011966204	CID000107722	CID000008956	CID000151438	CID005280644
CID025271599	CID000440384	CID000010634	CID070698354	CID005280720
CID011966172	CID000440460	CID000005880	CID000444899	CID005280933
CID000164800	CID053297338	CID000440368	CID000445049	CID005460412
CID024883423	CID011966205	CID000012594	CID005280360	CID006443013
CID011966175	CID003082089	CID000440573	CID005280363	CID006446027
CID000087642	CID005460041	CID000102030	CID000448457	CID009839084
CID000092153	CID000193321	CID000005756	CID005280383	CID005312830
CID000000743	CID071448894	CID000094141	CID005280427	CID005281128
CID000000984	CID046224536	CID000223997	CID005280492	CID009548877
CID003081383	CID046224539	CID000252379	CID005280493	CID010236635
CID000002682	CID005284270	CID046173741	CID005280497	CID025755601
CID005497143	CID003080603	CID000011302	CID005280701	CID005280411
CID009543037	CID005284271	CID000065542	CID005280724	CID005280729
CID000167759	CID000160520	CID000440623	CID005280733	CID005497123
CID000000757	CID000123743	CID000440624	CID005283170	CID005280934

CID011966146	CID046224544	CID000440625	CID005280745	CID005281166
CID011966194	CID000387316	CID000247304	CID005280778	CID005281167
CID011966165	CID000012544	CID000066414	CID005280876	CID005281929
CID000000096	CID025195379	CID000065554	CID005280877	CID005312508
CID000000180	CID000440985	CID000164838	CID005280878	CID005283028
CID000445127	CID000121948	CID000005864	CID005280879	CID005312889
CID000092135	CID011954196	CID000440694	CID005280880	CID000643941
CID011966129	CID000065094	CID000092745	CID005280881	CID016061072
CID000008215	CID011954197	CID000160499	CID005280883	CID007251182
CID000441449	CID000099470	CID000246873	CID005280884	CID006441679
CID000010466	CID015942889	CID000219833	CID005280885	CID006450029
CID000015868	CID044263831	CID000150891	CID005280886	CID000075704
CID000010459	CID000165511	CID000192735	CID005280888	CID006439873
CID005282922	CID003081084	CID000440707	CID005280889	CID006430107
CID000244872	CID000193396	CID000440708	CID005280890	CID016061034
CID000167775	CID003081085	CID000440709	CID005280892	CID023724728
CID000005997	CID003081958	CID000101788	CID005280893	CID005363388
CID000445713	CID015942888	CID021145112	CID000446284	CID000005951
CID000638072	CID024755558	CID000121935	CID000445580	CID005280335
CID005459811	CID050986116	CID006453841	CID005281116	CID000091486
CID000439423	CID000009903	CID000167685	CID000011197	CID000644260
CID000065728	CID000222528	CID000066416	CID005281120	CID005280458
CID005283731	CID000031401	CID000115255	CID025164091	CID000439853
CID005280453	CID005283821	CID000108192	CID056928001	CID005280613
CID000444679	CID005283820	CID000114833	CID056928013	CID005283560
CID000246983	CID000122340	CID000092786	CID056927904	CID000122121
CID000222284	CID005283831	CID000101771	CID056928012	CID000094140
CID000173183	CID005283852	CID009967418	CID056928006	CID005497111
CID000439577	CID000092805	CID006451493	CID056927909	CID003081439
CID000092110	CID005283845	CID003080558	CID070678705	CID005497112
CID000065252	CID005283892	CID000164674	CID056927910	CID005497113
CID000101690	CID000164853	CID000065076	CID000010467	CID011966132
CID011966190	CID056928014	CID005312529	CID005282768	CID000168166
CID071448924	CID056927911	CID006439848	CID005497181	CID003082227
ENSP00000263702	ENSP00000238651	ENSP00000325822	ENSP00000232892	ENSP00000363400
ENSP00000290429	ENSP00000323071	ENSP00000268053	ENSP00000299022	ENSP00000274793
ENSP00000280701	ENSP00000367086	ENSP00000264228	ENSP00000261292	ENSP00000259486
ENSP00000367462	ENSP00000357804	ENSP00000274192	ENSP00000358223	ENSP00000284116
ENSP00000258873	ENSP00000357807	ENSP00000354511	ENSP00000358232	ENSP00000321038
ENSP00000281455	ENSP00000250974	ENSP00000333310	ENSP00000337701	ENSP00000407375
ENSP00000350012	ENSP00000366240	ENSP00000201586	ENSP00000370430	ENSP00000374265
ENSP00000339787	ENSP00000258884	ENSP00000226444	ENSP00000256720	ENSP00000373477
ENSP00000348429	ENSP00000381654	ENSP00000312606	ENSP00000261596	ENSP00000392398
ENSP00000267842	ENSP00000363614	ENSP00000285930	ENSP00000362354	ENSP00000354581
ENSP00000209668	ENSP00000355308	ENSP00000338703	ENSP00000264775	ENSP00000354677
ENSP00000378359	ENSP00000373153	ENSP00000218516	ENSP00000329697	ENSP00000290349
ENSP00000296412	ENSP00000261875	ENSP00000389175	ENSP00000381302	ENSP00000290354
ENSP00000366927	ENSP00000357535	ENSP00000265276	ENSP00000392553	ENSP00000326219

ENSP00000345774	ENSP00000301729	ENSP00000352547	ENSP00000251473	ENSP00000251535
ENSP00000255084	ENSP00000346874	ENSP00000264409	ENSP00000352962	ENSP00000293761
ENSP00000387123	ENSP00000182377	ENSP00000380184	ENSP00000359204	ENSP00000369530
ENSP00000346827	ENSP00000228027	ENSP00000406674	ENSP00000292823	ENSP00000363512
ENSP00000261733	ENSP00000223114	ENSP00000198801	ENSP00000368439	ENSP00000354612
ENSP00000370023	ENSP00000363645	ENSP00000262134	ENSP00000260585	ENSP00000356438
ENSP00000296424	ENSP00000359297	ENSP00000310551	ENSP00000229266	ENSP00000221730
ENSP00000278353	ENSP00000254521	ENSP00000302177	ENSP00000219789	ENSP00000228740
ENSP00000231887	ENSP00000358421	ENSP00000337463	ENSP00000282541	ENSP00000248923
ENSP00000168216	ENSP00000265896	ENSP00000360761	ENSP00000301149	ENSP00000381340
ENSP00000369050	ENSP00000216862	ENSP00000291572	ENSP00000308610	ENSP00000292596
ENSP00000342007	ENSP00000228606	ENSP00000314036	ENSP00000255389	ENSP00000356859
ENSP00000332679	ENSP00000261507	ENSP00000285518	ENSP00000317300	ENSP00000295256
ENSP00000360317	ENSP00000264027	ENSP00000216180	ENSP00000261407	ENSP00000360687
ENSP00000260682	ENSP00000347717	ENSP00000328405	ENSP00000283415	ENSP00000345341
ENSP00000285979	ENSP00000279263	ENSP00000382260	ENSP00000355607	ENSP00000342385
ENSP00000360372	ENSP00000360316	ENSP00000264057	ENSP00000264005	ENSP00000262033
ENSP00000252945	ENSP00000356591	ENSP00000284061	ENSP00000337103	ENSP00000244043
ENSP00000333534	ENSP00000301466	ENSP00000265022	ENSP00000265689	ENSP00000308032
ENSP00000333212	ENSP00000220584	ENSP00000337572	ENSP00000384400	ENSP00000278840
ENSP00000222382	ENSP00000337354	ENSP00000288490	ENSP00000266517	ENSP00000306920
ENSP00000222982	ENSP00000348762	ENSP00000273814	ENSP00000356170	ENSP00000364782
ENSP00000337450	ENSP00000297679	ENSP00000351706	ENSP00000295887	ENSP00000233840
ENSP00000311095	ENSP00000301645	ENSP00000418001	ENSP00000419879	ENSP00000294064
ENSP00000360991	ENSP00000275016	ENSP00000368226	ENSP00000308258	ENSP00000367343
ENSP00000221700	ENSP00000310721	ENSP00000365725	ENSP00000368140	ENSP00000314508
ENSP00000221307	ENSP00000258415	ENSP00000268129	ENSP00000262764	ENSP00000307126
ENSP00000248041	ENSP00000318867	ENSP00000295962	ENSP00000289119	ENSP00000316476
ENSP00000321821	ENSP00000360918	ENSP00000379282	ENSP00000264735	ENSP00000271688
ENSP00000360968	ENSP00000307697	ENSP00000342793	ENSP00000320337	ENSP00000284382
ENSP00000334246	ENSP00000242375	ENSP00000263088	ENSP00000255688	ENSP00000251363
ENSP00000360958	ENSP00000360569	ENSP00000348901	ENSP00000301790	ENSP00000325485
ENSP00000265322	ENSP00000263093	ENSP00000376372	ENSP00000342026	ENSP00000262554
ENSP00000215567	ENSP00000343838	ENSP00000369099	ENSP00000312286	ENSP00000216484
ENSP00000293217	ENSP00000304845	ENSP00000266095	ENSP00000356436	ENSP00000381968
ENSP00000348775	ENSP00000418532	ENSP00000296486	ENSP00000396045	ENSP00000360776
ENSP00000242592	ENSP00000362508	ENSP00000264167	ENSP00000290472	ENSP00000306459
ENSP00000222214	ENSP00000362513	ENSP00000343234	ENSP00000215885	ENSP00000363397
ENSP00000409612	ENSP00000303174	ENSP00000262890	ENSP00000364249	ENSP00000216264
ENSP00000233710	ENSP00000362525	ENSP00000356015	ENSP00000364252	ENSP00000313681
ENSP00000285093	ENSP00000362549	ENSP00000265838	ENSP00000364246	ENSP00000245222
ENSP00000325136	ENSP00000346768	ENSP00000322706	ENSP00000364257	ENSP00000332656
ENSP00000333664	ENSP00000251566	ENSP00000358414	ENSP00000364243	ENSP00000219334
ENSP00000361536	ENSP00000305221	ENSP00000196371	ENSP00000393847	ENSP00000258052
ENSP00000346693	ENSP00000304811	ENSP00000361914	ENSP00000243501	ENSP00000261304
ENSP00000359022	ENSP00000274278	ENSP00000259407	ENSP00000330442	ENSP00000301452
ENSP00000358831	ENSP00000282507	ENSP00000217455	ENSP00000333142	ENSP00000342609
ENSP00000304736	ENSP00000265403	ENSP00000360366	ENSP00000257694	ENSP00000371152

ENSP00000265641	ENSP00000341045	ENSP00000311224	ENSP00000303211	ENSP00000378897
ENSP00000312189	ENSP00000320401	ENSP00000369927	ENSP00000325958	ENSP00000362298
ENSP00000319343	ENSP00000334276	ENSP00000381206	ENSP00000406909	ENSP00000359380
ENSP00000360541	ENSP00000358424	ENSP00000351035	ENSP00000368305	ENSP00000316329
ENSP00000363794	ENSP00000370254	ENSP00000364412	ENSP00000292427	ENSP00000358903
ENSP00000318631	ENSP00000199936			

4. Nucleotide metabolism

CID000005957	CID000439236	CID000006176	CID046173085	CID000068152
CID000006022	CID000073323	CID000006133	CID046173135	CID000160617
CID000000280	CID000439296	CID000000239	CID001751483	CID000439424
CID000000222	CID000000456	CID000006030	CID000000203	CID000443736
CID000006083	CID000024316	CID000001174	CID000013730	CID000006031
CID000008977	CID000439450	CID000006132	CID000006076	CID000008629
CID000000750	CID000021706	CID000001135	CID000439269	CID000006131
CID000006830	CID000165186	CID000005789	CID000015047	CID000066535
CID000000760	CID000439488	CID000000868	CID000000487	CID000148196
CID000010214	CID000146302	CID000013945	CID000000111	CID010290858
CID000159296	CID000041211	CID000000967	CID000440189	CID046173244
CID000001117	CID000000974	CID000006029	CID000065059	CID000000504
CID000005961	CID000064959	CID000439216	CID000001175	CID000091531
CID000008583	CID045479615	CID000164628	CID000001188	CID000165381
CID000001176	CID000439713	CID000009700	CID000006802	CID000250388
CID000006831	CID000439714	CID000065063	CID000065103	CID000160666
CID000439167	CID000000908	CID000000597	CID000000767	CID000000484
CID000007339	CID000439905	CID000000867	CID000006021	CID000065058
CID000008582	CID000161500	CID000000649	CID000445794	CID000000155
CID000015993	CID000014003	CID000093072	CID000187790	CID000000117
CID000006804	CID000447145	CID000065091	CID000012599	CID000440867
CID000000190	CID000160913	CID000064968	CID000439220	CID000008339
CID000000278	CID000009679	CID000065070	CID005287787	CID000166760
CID000188966	CID000130805	CID000006175	CID000145729	CID000165388
CID000000971	CID000038166	CID000013712	CID000101543	CID000150855
CID000060961	CID005462266	CID005460448	CID000000276	CID000006211
CID000010238	CID000065110	CID000000764	CID000000790	CID000013711
ENSP00000320171	ENSP00000362249	ENSP00000347046	ENSP00000307674	ENSP00000264424
ENSP00000339933	ENSP00000346921	ENSP00000255266	ENSP00000351520	ENSP00000261170
ENSP00000371393	ENSP00000346577	ENSP00000420295	ENSP00000335246	ENSP00000254854
ENSP00000361512	ENSP00000267584	ENSP00000360502	ENSP00000365840	ENSP00000218006
ENSP00000381504	ENSP00000298545	ENSP00000291539	ENSP00000370718	ENSP00000333019
ENSP00000345096	ENSP00000256722	ENSP00000332116	ENSP00000350265	ENSP00000355493
ENSP00000321584	ENSP00000219302	ENSP00000310661	ENSP00000369456	ENSP00000419851
ENSP00000300738	ENSP00000356785	ENSP00000311453	ENSP00000258662	ENSP00000313490
ENSP00000251810	ENSP00000013034	ENSP00000264917	ENSP00000382595	ENSP00000359211
ENSP00000259727	ENSP00000376886	ENSP00000330721	ENSP00000333490	ENSP00000219240
ENSP00000392859	ENSP00000219479	ENSP00000252505	ENSP00000297323	ENSP00000315644
ENSP00000371236	ENSP00000323036	ENSP00000262607	ENSP00000342952	ENSP00000264705
ENSP00000236959	ENSP00000235628	ENSP00000369099	ENSP00000260600	ENSP00000232607
ENSP00000354532	ENSP00000352904	ENSP00000419628	ENSP00000312126	ENSP00000330032

ENSP00000264220	ENSP00000339479	ENSP00000303575	ENSP0000419361	ENSP00000252029
ENSP00000298556	ENSP00000257770	ENSP00000355587	ENSP00000311405	ENSP00000301634
ENSP00000264093	ENSP00000243052	ENSP00000318066	ENSP00000254235	ENSP00000361289
ENSP00000265174	ENSP00000334910	ENSP00000339016	ENSP00000286355	ENSP00000356853
ENSP00000406157	ENSP00000351957	ENSP00000287394	ENSP00000294016	ENSP00000346155
ENSP00000371230	ENSP00000282096	ENSP00000294053	ENSP00000356825	ENSP00000304802
ENSP00000361699	ENSP00000286063	ENSP00000215862	ENSP00000357669	ENSP00000324343
ENSP00000352222	ENSP00000322524	ENSP00000301825	ENSP00000341083	ENSP00000276651
ENSP00000360561	ENSP00000296518	ENSP00000349576		

5. Amino acid metabolism

CID000000280	CID000000880	CID009833951	CID020802017	CID000439402
CID000000222	CID000000249	CID000115015	CID000006057	CID000005810
CID000033032	CID000000763	CID000006325	CID000000996	CID000193305
CID000000051	CID000439278	CID000000563	CID000006047	CID000108012
CID000005960	CID000000247	CID000001119	CID000005610	CID000107541
CID000006322	CID000071077	CID000443233	CID000000785	CID009548602
CID000005961	CID000000428	CID000443250	CID000000780	CID000069217
CID000006262	CID000068841	CID005462190	CID000439260	CID000000559
CID000001176	CID000000673	CID000000561	CID000003469	CID000122356
CID000444972	CID000151187	CID000161214	CID000000127	CID000018189
CID000000278	CID000000215	CID000444493	CID000005815	CID000188984
CID000009750	CID000439258	CID000001132	CID000637542	CID000193475
CID000439232	CID000152441	CID000092133	CID000439316	CID000439968
CID000070914	CID000000105	CID000092753	CID005280393	CID000005942
CID000150833	CID000134490	CID000006106	CID000009305	CID000127370
CID000192878	CID000440033	CID000000049	CID005280398	CID000440014
CID000016950	CID000099289	CID000000096	CID000000547	CID000440046
CID000440236	CID000126041	CID000006287	CID000000979	CID000000558
CID000001060	CID000441021	CID000000070	CID000444266	CID000440237
CID000000970	CID012011795	CID000092153	CID005459802	CID011966267
CID000005950	CID000034755	CID000000296	CID000005819	CID005280691
CID000001110	CID000439155	CID000445127	CID005281792	CID000440575
CID000006267	CID000124886	CID000006306	CID005280494	CID000440847
CID000000311	CID000001117	CID003036931	CID000009727	CID000440848
CID000001112	CID000006137	CID000439286	CID000005920	CID000123895
CID000000119	CID000001099	CID011966111	CID000439744	CID000440850
CID000440997	CID000091552	CID005287787	CID000000681	CID000012473
CID000083887	CID000439176	CID000449534	CID000440113	CID000092150
CID000093072	CID000000402	CID000000487	CID000119219	CID000440854
CID000000048	CID000000878	CID000165435	CID000119405	CID005281796
CID000065065	CID000067678	CID009549326	CID000440311	CID023724544
CID000439716	CID000072886	CID071448892	CID005280709	CID046174065
CID000439905	CID001549097	CID000439434	CID000091528	CID044229222
CID000447145	CID000092851	CID000193425	CID000151725	CID000007339
CID000440162	CID000000098	CID006443760	CID000114683	CID000006274
CID000071120	CID000099478	CID000165390	CID000440728	CID000000774
CID000000750	CID000439389	CID011966220	CID000085782	CID000439233
CID000000760	CID000439406	CID011966188	CID000151276	CID000736715

CID000005951	CID000439415	CID011966163	CID000001738	CID000165271
CID000006305	CID000000473	CID000440873	CID000440729	CID000088052
CID000005862	CID000000535	CID005462303	CID000001245	CID000439376
CID000091443	CID000010010	CID071448903	CID000070949	CID000064969
CID000000058	CID000165331	CID000643798	CID000001669	CID000112071
CID000000305	CID000123991	CID005280523	CID000688084	CID000000792
CID000439175	CID000080283	CID000439766	CID000688100	CID000152657
CID000000964	CID000158980	CID005462261	CID000012122	CID011988267
CID000006288	CID000439904	CID000005962	CID000010805	CID000096215
CID000439183	CID000439750	CID000000071	CID005280801	CID000000794
CID000001088	CID000440041	CID000160556	CID000161255	CID000440000
CID000439194	CID011988266	CID000439283	CID000010393	CID000000128
CID000000586	CID000174549	CID000099290	CID000159278	CID000440431
CID000000137	CID000000110	CID000092136	CID000068313	CID000065110
CID000439235	CID000440717	CID000439459	CID000348154	CID000193735
CID003037042	CID000000242	CID000194695	CID014178745	CID000003614
CID000440726	CID000444539	CID000440179	CID017796835	CID000150841
CID000193545	CID009543169	CID000160603	CID000006140	CID000069602
CID000075810	CID000165620	CID000440349	CID000000177	CID000000782
CID000121396	CID005280361	CID005459812	CID000000997	CID000000793
CID000164697	CID000000998	CID005460671	CID005371769	CID000068144
CID000439430	CID000000338	CID051055215	CID000351795	CID000643327
CID000439460	CID000009294	CID000122283	CID000011811	CID000101184
CID000000273	CID000000464	CID000439227	CID005280595	CID000000107
CID000092832	CID000637540	CID000000138	CID000001066	CID000011970
CID000439939	CID000011915	CID024771808	CID005280625	CID000006054
CID000439954	CID009543152	CID000000085	CID005280673	CID000000999
CID000439963	CID000071567	CID000000743	CID000074688	CID000000091
CID000440077	CID000007680	CID003081383	CID000001826	CID000637541
CID000440120	CID000007028	CID000057449	CID000164719	CID005282146
CID000128888	CID000074839	CID005497143	CID000171161	CID000011363
CID000193187	CID000000020	CID009543037	CID000001864	CID046926111
CID000001194	CID000092258	CID000000133	CID000440741	CID046926226
CID011966128	CID000001001	CID000000134	CID009548587	CID000000227
CID000123762	CID000439319	CID011966129	CID000101399	CID000161165
CID000443849	CID000439351	CID000000523	CID000050591	CID000000803
CID003032849	CID000439394	CID000000502	CID000001833	CID000001150
CID000001045	CID000439463	CID000145068	CID000018986	CID000000798
CID000145742	CID000439774	CID000000118	CID000440752	CID000000086
CID000000199	CID000121947	CID000001103	CID000076230	CID000000800
CID006440013	CID000444150	CID000008988	CID000151066	CID000439280
CID000001102	CID000160647	CID000000588	CID000656506	CID000005202
CID002724505	CID000007420	CID000000500	CID000006088	CID000000801
CID000001028	CID000005800	CID000439375	CID000006736	CID000010685
CID000122357	CID000011321	CID000000397	CID000001018	CID000000903
CID000006508	CID000005699	CID000439788	CID000001005	CID000000472
CID000172312	CID000008742	CID000021860	CID000000072	CID000000896
CID000012039	CID000003845			

ENSP00000327251	ENSP00000342032	ENSP00000347324	ENSP00000356015	ENSP00000373640
ENSP00000297494	ENSP00000313432	ENSP00000255192	ENSP00000265838	ENSP00000263556
ENSP00000277865	ENSP00000319851	ENSP00000355536	ENSP00000322706	ENSP00000166534
ENSP00000327589	ENSP00000387123	ENSP00000366156	ENSP00000358414	ENSP00000332170
ENSP00000293404	ENSP00000253799	ENSP00000385746	ENSP00000196371	ENSP00000360268
ENSP00000303077	ENSP00000312326	ENSP00000303147	ENSP00000361914	ENSP00000354193
ENSP00000359539	ENSP00000228476	ENSP00000322991	ENSP00000381654	ENSP00000328858
ENSP00000245206	ENSP00000340684	ENSP00000273920	ENSP00000363614	ENSP00000342502
ENSP00000378408	ENSP00000367309	ENSP00000315931	ENSP00000370023	ENSP00000307252
ENSP00000345282	ENSP00000370737	ENSP00000217426	ENSP00000357535	ENSP00000349577
ENSP00000317379	ENSP00000360938	ENSP00000357880	ENSP00000265395	ENSP00000368572
ENSP00000310447	ENSP00000255189	ENSP00000302227	ENSP00000363832	ENSP00000269298
ENSP00000357066	ENSP00000403536	ENSP00000260508	ENSP00000269980	ENSP00000299198
ENSP00000261783	ENSP00000361894	ENSP00000040663	ENSP0000318351	ENSP00000221476
ENSP00000307188	ENSP00000274353	ENSP00000366927	ENSP00000359151	ENSP00000254035
ENSP00000307900	ENSP00000318868	ENSP00000345774	ENSP00000352706	ENSP00000300283
ENSP00000402608	ENSP00000333667	ENSP00000255084	ENSP00000364883	ENSP00000226299
ENSP00000253004	ENSP00000273588	ENSP00000346827	ENSP00000244217	ENSP00000325548
ENSP00000264220	ENSP00000379895	ENSP00000261733	ENSP00000274813	ENSP00000351682
ENSP00000333019	ENSP00000309259	ENSP00000231887	ENSP00000324842	ENSP00000216271
ENSP00000355493	ENSP00000365773	ENSP00000168216	ENSP00000365462	ENSP00000364986
ENSP00000264705	ENSP00000389175	ENSP00000242592	ENSP00000265594	ENSP00000234111
ENSP00000314649	ENSP00000215904	ENSP00000409612	ENSP00000343657	ENSP00000359680
ENSP00000290597	ENSP00000275605	ENSP00000285093	ENSP00000226840	ENSP00000247194
ENSP00000357920	ENSP00000257549	ENSP00000325136	ENSP00000263035	ENSP00000225740
ENSP00000342557	ENSP00000341117	ENSP00000333664	ENSP00000222673	ENSP00000366030
ENSP00000354347	ENSP00000280706	ENSP00000360968	ENSP00000335304	ENSP00000280097
ENSP00000253778	ENSP00000302393	ENSP00000334246	ENSP00000222214	ENSP00000291670
ENSP00000268251	ENSP00000229319	ENSP00000241052	ENSP00000263182	ENSP00000266736
ENSP00000231420	ENSP00000280704	ENSP00000294973	ENSP00000196061	ENSP00000267845
ENSP00000302620	ENSP00000327070	ENSP00000250018	ENSP00000282903	ENSP00000261208
ENSP00000256999	ENSP00000348234	ENSP00000329093	ENSP00000223127	ENSP00000354511
ENSP00000301776	ENSP00000250535	ENSP00000265605	ENSP00000335261	ENSP00000370571
ENSP00000263080	ENSP00000333666	ENSP00000370639	ENSP00000377040	ENSP00000376776
ENSP00000377696	ENSP00000355084	ENSP00000013222	ENSP00000252599	ENSP00000263321
ENSP00000350928	ENSP00000365693	ENSP00000250615	ENSP00000354960	ENSP00000269582
ENSP00000259271	ENSP00000333212	ENSP00000264170	ENSP00000333534	ENSP00000261755
ENSP00000350136	ENSP00000222382	ENSP00000205402	ENSP00000221307	ENSP00000372112
ENSP00000414330	ENSP00000222982	ENSP00000359991	ENSP00000248041	ENSP00000392762
ENSP00000175506	ENSP00000337450	ENSP00000297283	ENSP00000321821	ENSP00000340200
ENSP00000209668	ENSP00000311095	ENSP00000412189	ENSP00000285979	ENSP00000369050
ENSP00000378359	ENSP00000360991	ENSP00000360317	ENSP00000360372	ENSP00000342007
ENSP00000296412	ENSP00000221700	ENSP00000260682	ENSP00000252945	ENSP00000332679

6. Metabolism of other amino acids

CID000444493	CID000005950	CID000119033	CID000013818	CID000003414
CID000005960	CID000001099	CID000000768	CID000000533	CID005462314
CID000644066	CID000005862	CID000000602	CID000011648	CID000151926
CID000000239	CID000000186	CID000011128	CID056928068	CID000162204

CID000092753	CID000001123	CID000006406	CID000105024	CID006337958
CID000001174	CID000072886	CID000440104	CID000001090	CID014647097
CID000006274	CID000107812	CID000161355	CID000025076	CID000007405
CID000000868	CID000160226	CID000656516	CID000147004	CID000449517
CID000001102	CID001549097	CID000441467	CID049787000	CID000115098
CID000000119	CID000006058	CID000008794	CID000001089	CID000440667
CID000000867	CID000068340	CID000005961	CID000441455	CID000065523
CID000000649	CID053297338	CID000023327	CID000440764	CID024892797
CID000000118	CID000007866	CID000449538	CID053297413	CID011020241
CID000001103	CID000068759	CID000145815	CID000091580	CID000065359
CID000010110	CID000000363	CID011006912	CID000161597	CID000123938
CID000006613	CID000031257	CID005496796	CID000000222	CID000439498
CID009543026	CID000000750	CID000006322	CID000005951	CID000000273
CID000000428	CID000001005	CID000006262	CID000006140	CID000000051
CID000068152	CID000000177	CID000001045	CID000006057	CID000000175
CID000076406	CID000439167	CID000071082	CID000006267	CID004369587
CID000112071	CID000000297	CID000012326	CID000006287	CID000073491
CID000000111	CID000439811	CID000439402	CID000000611	CID000097588
CID000041634	CID000000490	CID000440046	CID000006306	CID000000546
CID000001066	CID000000339	CID000000558	CID000000713	CID000089954
CID000000075	CID000091619	CID000005884	CID000001647	CID000001060
CID011966171	CID000003857	CID000005885	CID000124886	CID000033032
CID000001678	CID000440756			
ENSP00000406157	ENSP00000345023	ENSP00000366927	ENSP00000262430	ENSP00000256594
ENSP00000355536	ENSP00000300738	ENSP00000345774	ENSP00000259407	ENSP00000358851
ENSP00000302227	ENSP00000251810	ENSP00000255084	ENSP00000250535	ENSP00000256593
ENSP00000260508	ENSP00000234111	ENSP00000387123	ENSP00000362888	ENSP00000381607
ENSP00000013222	ENSP00000346103	ENSP00000346827	ENSP00000248923	ENSP00000290765
ENSP00000357882	ENSP00000262746	ENSP00000261733	ENSP00000381340	ENSP00000295256
ENSP00000367893	ENSP00000301522	ENSP00000225740	ENSP00000338964	ENSP00000233714
ENSP00000254663	ENSP00000298510	ENSP00000293217	ENSP00000292823	ENSP00000010404
ENSP00000262027	ENSP00000368646	ENSP00000348775	ENSP00000368439	ENSP00000265498
ENSP00000282276	ENSP00000265462	ENSP00000242592	ENSP00000260585	ENSP00000226299
ENSP00000301776	ENSP00000342026	ENSP00000231887	ENSP00000229266	ENSP00000300060
ENSP00000318868	ENSP00000253063	ENSP00000370023	ENSP00000265174	ENSP00000295304
ENSP00000333667	ENSP00000407375	ENSP00000357535	ENSP00000358727	ENSP00000275428
ENSP00000277865	ENSP00000374265	ENSP00000359211	ENSP00000335620	ENSP00000385746
ENSP00000327589	ENSP00000373477	ENSP00000324343	ENSP00000420168	ENSP00000352706
ENSP00000317379	ENSP00000392398	ENSP00000276651	ENSP00000211122	ENSP00000307252
ENSP00000310447	ENSP00000354581	ENSP00000268251	ENSP00000359998	ENSP00000325548
ENSP00000228476	ENSP00000354677	ENSP00000350928	ENSP00000284562	ENSP00000351682
ENSP00000260985	ENSP00000221130	ENSP00000259271	ENSP00000311469	ENSP00000267085
ENSP00000331897	ENSP00000360688	ENSP00000253799	ENSP00000241337	ENSP00000282538
ENSP00000270776	ENSP00000272425	ENSP00000312326	ENSP00000215780	ENSP00000366156
ENSP00000377192				

7. Glycan biosynthesis and metabolism

CID000018396	CID000439184	CID024892725	CID000165130	CID005496796
CID024892715	CID000165007	CID024892739	CID000151104	CID005280700

CID024892719	CID000449538	CID000445070	CID000010214	CID000644109
CID024892721	CID011006912	CID000006176	CID000159296	CID000121966
CID000445675	CID000000175			
ENSP00000264228	ENSP00000320965	ENSP00000317027	ENSP00000354960	ENSP00000268695
ENSP00000311888	ENSP00000353910	ENSP00000223122	ENSP00000361302	ENSP00000308107
ENSP00000239891	ENSP00000360644	ENSP00000269195	ENSP00000261534	ENSP00000220244
ENSP00000417764	ENSP00000169298	ENSP00000355632	ENSP00000322300	ENSP00000266031
ENSP00000262374	ENSP00000355273	ENSP00000376465	ENSP00000266712	ENSP00000350387
ENSP00000307423	ENSP00000376472	ENSP00000259056	ENSP00000364902	ENSP00000223026
ENSP00000345270	ENSP00000295770	ENSP00000348668	ENSP00000339613	ENSP00000345849
ENSP00000264968	ENSP00000361667	ENSP00000265000	ENSP00000222725	ENSP00000247933
ENSP00000338487	ENSP00000318147	ENSP00000252318	ENSP00000349490	ENSP00000029410
ENSP00000281923	ENSP00000357453	ENSP00000380488	ENSP00000307971	ENSP00000368496
ENSP00000391227	ENSP00000348959	ENSP00000297107	ENSP00000295571	ENSP00000363590
ENSP00000266483	ENSP00000360645	ENSP00000364150	ENSP00000362824	ENSP00000254190
ENSP00000310120	ENSP00000363452	ENSP00000376570	ENSP00000385478	ENSP00000243776
ENSP00000380793	ENSP00000261483	ENSP00000288988	ENSP00000307875	ENSP00000302629
ENSP00000333813	ENSP00000353655	ENSP00000344260	ENSP00000230053	ENSP00000035307
ENSP00000299626	ENSP00000340466	ENSP00000336729	ENSP00000265471	ENSP00000295588
ENSP00000369055	ENSP00000306920	ENSP00000329654	ENSP00000344125	ENSP00000365172
ENSP00000369820	ENSP00000364782	ENSP00000227756	ENSP00000355559	ENSP00000261381
ENSP00000216452	ENSP00000233840	ENSP00000156626	ENSP00000331258	ENSP00000017003
ENSP00000420037	ENSP00000294064	ENSP00000225276	ENSP00000302728	ENSP00000362207
ENSP00000379680	ENSP00000395473	ENSP00000345477	ENSP00000369442	ENSP00000276055
ENSP00000390784	ENSP00000285599	ENSP00000227495	ENSP00000264914	ENSP00000333947
ENSP00000341562	ENSP00000267978	ENSP00000266383	ENSP00000230036	ENSP00000305725
ENSP00000329214	ENSP00000367343	ENSP00000328277	ENSP00000225609	ENSP00000258711
ENSP00000367446	ENSP00000314508	ENSP00000305603	ENSP00000265012	ENSP00000317404
ENSP00000002596	ENSP00000002165	ENSP00000252675	ENSP00000316173	ENSP00000305595
ENSP00000330606	ENSP00000363603	ENSP00000286955	ENSP00000249005	ENSP00000321874
ENSP00000261374	ENSP00000268097	ENSP00000262915	ENSP00000352144	ENSP00000319636
ENSP00000284110	ENSP00000261416	ENSP00000223127	ENSP00000375748	ENSP00000309270
ENSP00000354213	ENSP00000337854	ENSP00000252599	ENSP00000323479	ENSP00000359131
ENSP00000264595	ENSP00000356404	ENSP00000220562	ENSP00000379353	ENSP00000218516
ENSP00000365920				

8. Metabolism of cofactors and vitamins

CID000006057	CID005280839	CID000440901	CID010261389	CID000119055
CID000000135	CID005280845	CID000193577	CID000115084	CID000442163
CID006440013	CID000441209	CID000000005	CID000000437	CID000128973
CID000012039	CID011954010	CID000005885	CID000579061	CID000169508
CID000445995	CID011954011	CID000001110	CID000000434	CID000000666
CID000447277	CID000092094	CID000005960	CID057339263	CID000065074
CID005280346	CID005282350	CID000000668	CID000087642	CID000121885
CID000444539	CID044140569	CID000444972	CID000159296	CID000440840
CID000000780	CID023724601	CID000000936	CID000000239	CID000444331
CID000637542	CID023724602	CID000001032	CID000001174	CID000448839
CID005280374	CID024883454	CID000000937	CID000000049	CID000440843
CID000189062	CID010364451	CID000000119	CID000006287	CID000443142

CID000000979	CID000479503	CID000014180	CID000000649	CID046173804
CID005280413	CID005462210	CID000165490	CID000439251	CID000446357
CID005280473	CID046173938	CID000005570	CID000439322	CID000000171
CID005280483	CID046173940	CID000072924	CID000006613	CID016061579
CID000014985	CID021587070	CID000439383	CID000444485	CID045266867
CID000092729	CID010902296	CID000099456	CID000000038	CID053356704
CID000000955	CID010946654	CID000121991	CID000115254	CID012463319
CID000168718	CID005283544	CID000439476	CID000000111	CID013344694
CID071448891	CID005283546	CID000444266	CID000041634	CID010220406
CID005280585	CID044237185	CID005280451	CID000440217	CID000638015
CID000000671	CID013850222	CID000439700	CID000440304	CID000445354
CID006443777	CID000091443	CID000000457	CID000131204	CID000444795
CID005280650	CID000439175	CID000095155	CID049859611	CID005280382
CID005280651	CID000122347	CID000439886	CID000005962	CID005280489
CID005280760	CID000098792	CID000069371	CID000001176	CID005280490
CID005280776	CID009825434	CID000439924	CID000171548	CID005280531
CID014556929	CID000439237	CID000440020	CID000000652	CID005459829
CID005280826	CID000006037	CID000001066	CID003082140	CID005281877
CID005280827	CID000000530	CID000068499	CID000000173	CID000446798
CID005280828	CID000000143	CID000000796	CID071448890	CID000449171
CID005280829	CID000005892	CID000161233	CID000000643	CID006438629
CID005280831	CID000001051	CID000069698	CID000000385	CID006437063
CID005280832	CID000001060	CID000440810	CID000440721	CID005363137
CID005280834	CID000000750	CID000440932	CID005326875	CID006436082
CID005280835	CID000001132	CID054723871	CID000086492	CID005289090
CID005280836	CID000005862	CID000070703	CID000083833	CID000033032
CID005280837	CID000439168	CID000000942	CID000083863	CID000004973
CID005287432	CID000001130	CID054693777	CID023724672	CID000006288
CID005280352	CID000001131	CID000001050	CID000006112	CID024892716
CID005280353	CID000000777	CID000000756	CID000007339	CID000000137
CID046173705	CID000000511	CID000122357	CID000133246	CID046173785
CID000439303	CID000161500	CID000001054	CID000044257	CID023724514
CID000001021	CID000001136	CID000001052	CID000000978	CID023724516
CID024836902	CID000001137	CID000001055	CID000065253	CID011953957
CID000000788	CID000000216	CID000001053	CID000000170	CID005281957
CID000001179	CID000000217	CID000006723	CID005462234	CID000443429
CID000121893	CID000443201	CID000151228	CID000000218	CID003246323
CID000439664	CID003080609	CID000439464	CID000196146	CID000023925
CID000004971	CID000597195	CID000449304	CID005460020	CID015719509
CID005280516	CID046942369	CID000440474	CID000196427	CID015775275
CID000072423	CID000066762	CID000493570	CID000008434	CID005323510
CID000119412	CID000643975	CID000006912	CID005280816	CID046174034
CID000439833	CID000006830	CID014080393	CID005280817	CID023657843
CID000000321	CID000643976	CID000446013	CID000026818	CID053297369
CID000440035	CID000439184	CID005326566	CID005280818	CID046906047
CID000129297	CID000114935	CID000000711	CID005280819	CID011953882
CID000440377	CID011953856	CID000000675	CID011953875	CID000068271
CID000440775	CID046173745	CID000168989	CID005280865	CID005280930

CID000440776	CID000160433	CID000001112		
ENSP00000378426	ENSP00000292180	ENSP00000239231	ENSP00000346768	ENSP00000264613
ENSP00000353998	ENSP00000365773	ENSP00000367727	ENSP00000251566	ENSP00000273550
ENSP00000319788	ENSP00000363832	ENSP00000278618	ENSP00000305221	ENSP00000313691
ENSP00000254759	ENSP00000225573	ENSP00000356905	ENSP00000304811	ENSP00000265523
ENSP00000288532	ENSP00000291565	ENSP00000322276	ENSP00000274278	ENSP00000343943
ENSP00000348234	ENSP00000215904	ENSP00000343190	ENSP00000282507	ENSP00000261643
ENSP00000233838	ENSP00000352782	ENSP00000280701	ENSP00000265403	ENSP00000246337
ENSP00000371236	ENSP00000354532	ENSP00000338387	ENSP00000341045	ENSP00000386284
ENSP00000236959	ENSP00000235628	ENSP00000309463	ENSP00000320401	ENSP00000357775
ENSP00000315644	ENSP00000352904	ENSP00000285930	ENSP00000334276	ENSP00000326579
ENSP00000318868	ENSP00000339479	ENSP00000370571	ENSP00000369050	ENSP00000355890
ENSP00000333667	ENSP00000257770	ENSP00000250018	ENSP00000342007	ENSP00000265512
ENSP00000273588	ENSP00000314649	ENSP00000329093	ENSP00000332679	ENSP00000307607
ENSP00000355536	ENSP00000373860	ENSP00000290349	ENSP00000360317	ENSP00000258168
ENSP00000291670	ENSP00000299964	ENSP00000290354	ENSP00000260682	ENSP00000334128
ENSP00000379108	ENSP00000222553	ENSP00000326219	ENSP00000285979	ENSP00000297785
ENSP00000377617	ENSP00000378782	ENSP00000234454	ENSP00000360372	ENSP00000249750
ENSP00000365775	ENSP00000226279	ENSP00000281243	ENSP00000252945	ENSP00000332256
ENSP00000319170	ENSP00000354387	ENSP00000261326	ENSP00000333534	ENSP00000295802
ENSP00000377083	ENSP00000168977	ENSP00000378890	ENSP00000333212	ENSP00000337224
ENSP00000258874	ENSP00000287713	ENSP00000254908	ENSP00000222382	ENSP00000228027
ENSP00000356290	ENSP00000340523	ENSP00000280362	ENSP00000222982	ENSP00000262340
ENSP00000362249	ENSP00000265016	ENSP00000362344	ENSP00000337450	ENSP00000302728
ENSP00000346921	ENSP00000230792	ENSP00000209668	ENSP00000311095	ENSP00000369442
ENSP00000346577	ENSP00000401508	ENSP00000378359	ENSP00000360991	ENSP00000309259
ENSP00000267584	ENSP00000326424	ENSP00000296412	ENSP00000221700	ENSP00000216117
ENSP00000298545	ENSP00000359211	ENSP00000343838	ENSP00000221307	ENSP00000219700
ENSP00000355587	ENSP00000324343	ENSP00000304845	ENSP00000248041	ENSP00000350265
ENSP00000353165	ENSP00000276651	ENSP00000418532	ENSP00000321821	ENSP00000369456
ENSP00000363205	ENSP00000322991	ENSP00000362508	ENSP00000360968	ENSP00000258662
ENSP00000375881	ENSP00000267085	ENSP00000362513	ENSP00000334246	ENSP00000263368
ENSP00000295463	ENSP00000282538	ENSP00000303174	ENSP00000381206	ENSP00000270593
ENSP00000295453	ENSP00000302108	ENSP00000362525	ENSP00000318631	ENSP00000288014
ENSP00000363965	ENSP00000313377	ENSP00000362549	ENSP00000306606	ENSP00000370718
ENSP00000327557	ENSP00000218758	ENSP00000272065	ENSP00000256997	ENSP00000323036

9. Metabolism of terpenoids and polyketides

CID000644066	CID000010582	CID000441005	CID016667385	CID000189062
CID000638011	CID005281994	CID000003503	CID000029746	CID000006057
CID000637566	CID010953718	CID000073170	CID006365330	CID000000979
CID009549320	CID000173183	CID000073145	CID009855795	CID023724518
CID009549326	CID000115196	CID000259846	CID005460659	CID000443709
CID000643820	CID011988279	CID000092097	CID011241545	CID000443710
CID005275520	CID023724579	CID000161937	CID025244915	CID010100825
CID000010402	CID023724581	CID005281517	CID056928117	CID006436726
CID009549319	CID023724582	CID005281519	CID044237280	CID006447533
CID009549328	CID023724583	CID005281520	CID056928122	CID000000019
CID000092753	CID023724584	CID000442393	CID025245907	CID000012039

CID003036931	CID023724585	CID014355861	CID010895555	CID000000338
CID011966111	CID023724586	CID011052747	CID056928123	CID071448900
CID000441110	CID023724590	CID000122283	CID006476390	CID000443765
CID046173788	CID023724591	CID000643976	CID010003607	CID000444493
CID000441109	CID023724592	CID000000711	CID012313974	CID000369312
CID000065533	CID023724593	CID000439279	CID024873746	CID000031253
CID000443211	CID023724594	CID000002758	CID056927728	CID000440917
CID000443210	CID046173984	CID000442495	CID025001002	CID006440982
CID000121966	CID046173985	CID000016724	CID056927810	CID010522005
CID016757114	CID056928129	CID000443158	CID012302184	CID023724755
CID024892770	CID005280489	CID000443178	CID025203718	CID000092180
CID000439292	CID006449797	CID011073781	CID000005997	CID000100221
CID000441096	CID000005957	CID000016441	CID000019212	CID000061020
CID000008369	CID000006022	CID000006654	CID000439423	CID000688597
CID025150860	CID000006031	CID000330573	CID000439743	CID005280589
CID056927715	CID000034755	CID013935024	CID005281523	CID023724748
CID056927946	CID000006083	CID000638072	CID015942885	CID023724749
CID000447277	CID000008629	CID005459811	CID014017685	CID000024405
CID000445713	CID000000175	CID000194140	CID013939876	CID011561034
CID000445070	CID000000190	CID000114857	CID023724769	CID011132720
CID005280413	CID000439176	CID000656496	CID005275507	CID010357552
CID005280598	CID023724459	CID000168951	CID005275508	CID021896400
CID005280650	CID000000647	CID000164874	CID015081546	CID000445995
CID005280651	CID000449093	CID000092155	CID005459948	CID000439250
CID005280700	CID000099478	CID000000355	CID006324616	CID000026447
CID014556929	CID005461055	CID000445805	CID000127115	CID000094221
CID005462210	CID000194139	CID015519422	CID000443628	CID000161276
CID006438372	CID005461146	CID000036143	CID046173796	CID000439711
ENSP00000231887	ENSP00000366927	ENSP00000358414	ENSP00000228510	ENSP00000356015
ENSP00000168216	ENSP00000345774	ENSP00000287936	ENSP00000357452	ENSP00000265838
ENSP00000285093	ENSP00000255084	ENSP00000387654	ENSP00000361845	ENSP00000322706
ENSP00000325136	ENSP00000387123	ENSP00000343552	ENSP00000301012	ENSP00000220584
ENSP00000333664	ENSP00000346827	ENSP00000349078	ENSP00000370748	ENSP00000358931
ENSP00000381654	ENSP00000261733	ENSP00000282841	ENSP00000277517	ENSP00000280605
ENSP00000363614	ENSP00000264228	ENSP00000303423	ENSP00000260682	ENSP00000358033
ENSP00000370023	ENSP00000274192	ENSP00000246166	ENSP00000360372	ENSP00000365388
ENSP00000357535	ENSP00000321810	ENSP00000353104	ENSP00000357480	ENSP00000265896
ENSP00000261296				

10. Biosynthesis of other secondary metabolites

CID000000760	CID000006287	CID011953794	CID005281426	CID005281672
CID000001188	CID000065536	CID000439440	CID000637563	CID005280362
CID000064959	CID000092136	CID000439467	CID000008815	CID005280417
CID000002153	CID000011082	CID000121966	CID000003314	CID005280442
CID000005429	CID000440722	CID024892737	CID000007127	CID000162350
CID000002519	CID000440723	CID000440194	CID000853433	CID005280459
CID000004687	CID000071724	CID000439292	CID000637776	CID005280637
CID000068374	CID000160139	CID000008629	CID005281871	CID005280666
CID000091611	CID000441084	CID000445675	CID044229078	CID005280681

CID000070639	CID000441328	CID000036294	CID005282094	CID005280682
CID000080220	CID000446570	CID000169075	CID005282095	CID005280746
CID000069726	CID000439168	CID000072396	CID005282096	CID005280804
CID000079437	CID000087642	CID000072392	CID000068148	CID005280805
CID000181740	CID000033032	CID003081544	CID014777879	CID005281666
CID000011719	CID000145742	CID000008378	CID015241070	CID005281953
CID000007293	CID000439322	CID000033042	CID046173191	CID005282102
CID000108214	CID000115254	CID000006032	CID044237218	CID005282150
CID000088299	CID000193305	CID000203442	CID005280343	CID005282152
CID000444493	CID000193475	CID000072395	CID005280441	CID005282154
CID000644066	CID000440162	CID023724518	CID005280443	CID005282155
CID005280389	CID000441128	CID010583108	CID005280445	CID005316673
CID000104940	CID000441133	CID000194428	CID005280863	CID000006047
CID002724360	CID000003893	CID046173948	CID000160487	CID000005610
CID020832874	CID000001028	CID000123865	CID000005154	CID000002353
CID021596453	CID054675768	CID000072394	CID005459910	CID005288826
CID000025102	CID015940185	CID000197189	CID005460444	CID005459823
CID046926144	CID054706138	CID016757114	CID005324289	CID000072309
CID000034755	CID054691343	CID000041774	CID005284371	CID000439653
CID000001060	CID054691348	CID000443629	CID005462306	CID000072301
CID000005960	CID000443861	CID000000887	CID000159911	CID000021171
CID000001117	CID000000798	CID000001150	CID000441066	CID000000681
CID000006322	CID000444150	CID000161276	CID000441069	CID000440113
CID000005951	CID000447277	CID000006140	CID000004680	CID000119219
CID000006057	CID000006305	CID006440013	CID000441257	CID000440583
CID000006288	CID000000227	CID000001102	CID000441259	CID000440584
CID000010238	CID000012039	CID011966126	CID000006167	CID000440586
CID000439235	CID000445344	CID011966129	CID000009651	CID005408233
CID000000979	CID000005793	CID011966120	CID000441593	CID000004970
CID005460143	CID000005958	CID000444539	CID000072378	CID005460042
CID006400657	CID000065533	CID000637775	CID000010219	CID000019009
CID000152441	CID000000892	CID006444037	CID000275196	CID000440927
CID000000355	CID000019648	CID001549095	CID000442330	CID005280555
CID000440179	CID000443211	CID005280372	CID000443421	CID000641301
CID000036143	CID000439294	CID000637542	CID000165003	CID005280802
CID025201122	CID000443210	CID001794426	CID000398937	CID000446834
CID000005862	CID000439408	CID000637511	CID009838394	CID000062453
CID000079038	CID000160603	CID005280385	CID000008768	CID000000323
CID000446220	CID000001194	CID000689043	CID000160500	CID000000368
CID000010726	CID000643327	CID000445858	CID000005962	CID000000412
CID000174174	CID005280906	CID000439514	CID000001045	CID000101615
CID000000273	CID000010198	CID005316860	CID000000997	CID000011388
CID000008082	CID000440932	CID005280460	CID000000937	CID000051683
CID000154417	CID000002181	CID000637540	CID000006306	CID000644020
CID000439791	CID000068161	CID005280507	CID000439227	CID000188288
CID000439954	CID049787007	CID005280535	CID024771808	CID000443848
CID000638024	CID000439316	CID005280536	CID000008424	CID000443849
CID000006106	CID000001669	CID011953815	CID000089594	CID005380876

CID000000049	CID000161255	CID005371769	CID000000051	CID024883456
CID000000070	CID025245127	CID003295299	CID000000970	CID025203496
CID000439286	CID000162602	CID000656506	CID000000750	CID011298145
CID000000473	CID000147311	CID000656524	CID000643976	CID000228215
CID000656497	CID000006137	CID000656534	CID000006262	CID022266650
CID000099478	CID000005950	CID000656538	CID000000311	CID002724505
CID000068841	CID003036931	CID000656567	CID000439183	CID046173877
CID000123895	CID000001183	CID000194140	CID000009750	CID000445713
CID000071082				
ENSP00000369050	ENSP00000311095	ENSP00000348234	ENSP00000252804	ENSP00000225371
ENSP00000342007	ENSP00000360991	ENSP00000303077	ENSP00000302728	ENSP00000262290
ENSP00000332679	ENSP00000221700	ENSP00000359539	ENSP00000369442	ENSP00000348645
ENSP00000360317	ENSP00000221307	ENSP00000245206	ENSP00000342557	ENSP00000222982
ENSP00000260682	ENSP00000248041	ENSP00000346643	ENSP00000253799	ENSP00000337450
ENSP00000285979	ENSP00000321821	ENSP00000290573	ENSP00000312326	ENSP00000228476
ENSP00000360372	ENSP00000360968	ENSP00000292432	ENSP00000340684	ENSP00000360268
ENSP00000252945	ENSP00000334246	ENSP00000223366	ENSP00000367309	ENSP00000269159
ENSP00000333534	ENSP00000286479	ENSP00000371393	ENSP00000370571	ENSP00000261296
ENSP00000333212	ENSP00000265174	ENSP00000262644	ENSP00000263321	ENSP00000322991
ENSP00000222382	ENSP00000406157	ENSP00000408526	ENSP00000354511	

11. Xenobiotics biodegradation and metabolism

CID000000280	CID000005054	CID000000569	CID000441164	CID000000174
CID000000996	CID009543152	CID000003035	CID000007929	CID000006326
CID005280366	CID054675756	CID000003036	CID000012105	CID000006344
CID000001060	CID000007478	CID000016323	CID000007418	CID000006325
CID000444493	CID000168718	CID000007095	CID000007237	CID000006354
CID000000177	CID005280595	CID000015625	CID000006994	CID000024726
CID000000289	CID000000104	CID000000568	CID000010722	CID000643794
CID000092133	CID000176951	CID000009216	CID000008373	CID000000011
CID000000242	CID000006926	CID000006854	CID000011476	CID000000034
CID000000019	CID000440983	CID000036187	CID000000072	CID000000033
CID000092153	CID000007413	CID000063090	CID000000240	CID000000300
CID003081383	CID024798714	CID000038439	CID009543169	CID000031373
CID000000785	CID000000135	CID000038251	CID000000244	CID000006575
CID005280361	CID000000126	CID000042540	CID000007420	CID000638186
CID000000093	CID000007469	CID000029575	CID000001140	CID000643833
CID005497143	CID000006618	CID000039929	CID000000342	CID000006338
CID009543037	CID000080801	CID000042128	CID000002879	CID000008259
CID000000241	CID000006623	CID000051130	CID000000101	CID000028167
CID000008404	CID000031242	CID000038199	CID000440385	CID000006597
CID000011390	CID005280500	CID000051043	CID000440399	CID000006421
CID000007322	CID000006318	CID000036231	CID000006101	CID000006407
CID000006812	CID000000051	CID000036188	CID011966300	CID000006278
CID000008420	CID000000227	CID000053036	CID000007771	CID000006365
CID000006115	CID000000338	CID000035823	CID009543293	CID000024472
CID000004650	CID000000254	CID000047650	CID009543294	CID000003469
CID000000978	CID000062348	CID000038019	CID009543296	CID000000931
CID000000086	CID000008479	CID000050891	CID025021177	CID000074128

CID000001183	CID000000991	CID000040479	CID000011318	CID000440294
CID000000980	CID000000220	CID000018636	CID000006844	CID000006998
CID000001057	CID000009395	CID000092753	CID005280688	CID005280911
CID000000370	CID000000655	CID000000527	CID000007489	CID000000584
CID000000460	CID000000654	CID000007470	CID000002336	CID000007002
CID000011914	CID000008468	CID000000340	CID000000760	CID000006738
CID000439616	CID000189003	CID000007463	CID000000283	CID000020908
CID000005800	CID005280981	CID000029587	CID000000712	CID000006195
CID000011915	CID009543219	CID000009958	CID000000757	CID000007055
CID000000359	CID000007416	CID000007809	CID000000971	CID000006201
CID003505109	CID000011473	CID000011505	CID000000868	CID000007123
CID000000403	CID000012511	CID000007725	CID000000281	CID000005788
CID000007518	CID000007505	CID000066827	CID015558498	CID000007500
CID000012793	CID000002331	CID000011087	CID000006001	CID000007410
CID000010787	CID000010742	CID009543331	CID000011285	CID009543204
CID000000378	CID000011137	CID000683563	CID000007444	CID000068490
CID000000355	CID000000998	CID054731235	CID004660280	CID000443135
CID000000372	CID003081970	CID000016487	CID000069212	CID000444972
CID005280625	CID009543026	CID015608168	CID000070758	CID000000096
CID005280679	CID005280393	CID005462303	CID000001032	CID000107689
CID000019829	CID005280398	CID000008461	CID000007501	CID000006581
CID000151426	CID000006579	CID000007261	CID009543332	CID000000780
CID000001176	CID000007855	CID000008376	CID000089594	CID011094749
CID000150833	CID000007680	CID000029574	CID000008530	CID000008640
CID000007967	CID000012122	CID000037182	CID000000362	CID011954051
CID000007965	CID000011970	CID000006593	CID000002707	CID011954053
CID000007966	CID000000999	CID003036914	CID000007961	CID000108121
CID000000564	CID000008794	CID000105131	CID000007839	CID003036760
CID000007533	CID000115084	CID000047289	CID000008663	CID000007244
CID000092886	CID000108081	CID000004095	CID000007005	CID000037456
CID000000016	CID000000437	CID000003121	CID000006366	CID000037455
CID000440918	CID000104856	CID000034312	CID000006849	CID000037786
CID000014490	CID000000438	CID000083852	CID000007808	CID000025892
CID000000196	CID000104757	CID000002555	CID000028598	CID000041322
CID000013006	CID000104756	CID000003331	CID000010667	CID011954069
CID000007768	CID053297444	CID000002771	CID000011305	CID000119521
CID000008078	CID000156652	CID000099735	CID000155957	CID000006576
CID000013017	CID000183009	CID000033676	CID006378383	CID000006577
CID000079129	CID000007847	CID000107744	CID010090750	CID011954070
CID005489029	CID005288826	CID000031515	CID000114861	CID011954072
CID005360621	CID000449459	CID000096356	CID000009533	CID011954073
CID021350391	CID005284371	CID000002907	CID000099303	CID000121997
CID000010312	CID000002554	CID000015130	CID000124143	CID000151244
CID000129274	CID000003690	CID005462508	CID000161824	CID000013350
CID013103864	CID000003676	CID003036580	CID000024415	CID000009427
CID000162180	CID002733525	CID003033895	CID000087833	CID000150856
CID000162976	CID002723601	CID000091477	CID000076543	CID010037499
CID010403174	CID000003385	CID000222865	CID003015693	CID000060837

CID009944000	CID000104842	CID000005881	CID009925873	CID000005997
CID005484731	CID000443154	CID000440483	CID000667490	CID000006128
CID000104896	CID000005790	CID000441302	CID000008642	CID000006013
CID000134466	CID000060953	CID000013472	CID003034391	CID003081384
CID000135243	CID000005386	CID000160531	CID000009321	CID003034646
CID000134459	CID000018343	CID000134970	CID000005236	CID000018209
CID000173719	CID010077584	CID000005892	CID000002265	CID000014039
CID006437068	CID011756356	CID000005885	CID000003767	CID000071602
CID024892812	CID003032417	CID000005778	CID000005922	CID010091038
CID003731686	CID003031829			
ENSP00000231887	ENSP00000248041	ENSP00000298545	ENSP00000233714	ENSP00000333212
ENSP00000168216	ENSP00000321821	ENSP00000256722	ENSP00000010404	ENSP00000311095
ENSP00000222214	ENSP00000360968	ENSP00000219302	ENSP00000265498	ENSP00000284562
ENSP00000285093	ENSP00000334246	ENSP00000356785	ENSP00000290349	ENSP00000359998
ENSP00000325136	ENSP00000238618	ENSP00000013034	ENSP00000290354	ENSP00000333534
ENSP00000333664	ENSP00000378161	ENSP00000376886	ENSP00000326219	ENSP00000252945
ENSP00000356015	ENSP00000286479	ENSP00000219479	ENSP00000369927	ENSP00000211122
ENSP00000265838	ENSP00000261755	ENSP00000419851	ENSP00000370254	ENSP00000221307
ENSP00000370023	ENSP00000312606	ENSP00000359211	ENSP00000221403	ENSP00000337450
ENSP00000357535	ENSP00000225740	ENSP00000232607	ENSP00000370129	ENSP00000360372
ENSP00000219054	ENSP00000343838	ENSP00000330032	ENSP00000222002	ENSP00000420168
ENSP00000327453	ENSP00000304845	ENSP00000252029	ENSP00000272167	ENSP00000221700
ENSP00000301956	ENSP00000418532	ENSP00000301634	ENSP00000340684	ENSP00000222982
ENSP00000222381	ENSP00000362508	ENSP00000361289	ENSP00000367309	ENSP00000285979
ENSP00000222572	ENSP00000362513	ENSP00000356853	ENSP00000363832	ENSP00000335620
ENSP00000265627	ENSP00000303174	ENSP00000346155	ENSP00000346901	ENSP00000360991
ENSP00000209668	ENSP00000362525	ENSP00000324343	ENSP00000209929	ENSP00000222382
ENSP00000378359	ENSP00000362549	ENSP00000276651	ENSP00000356729	ENSP00000260682
ENSP00000296412	ENSP00000346768	ENSP00000353720	ENSP00000356723	ENSP00000215780
ENSP00000366927	ENSP00000251566	ENSP00000317842	ENSP00000254090	ENSP00000295256
ENSP00000345774	ENSP00000305221	ENSP00000304782	ENSP00000312304	ENSP00000267584
ENSP00000255084	ENSP00000304811	ENSP00000358421	ENSP00000302728	ENSP00000360317
ENSP00000387123	ENSP00000274278	ENSP00000358424	ENSP00000369442	ENSP00000345023
ENSP00000346827	ENSP00000282507	ENSP00000311469	ENSP00000345096	ENSP00000290765
ENSP00000261733	ENSP00000265403	ENSP00000241337	ENSP00000321584	ENSP00000346577
ENSP00000221730	ENSP00000341045	ENSP00000256594	ENSP00000300738	ENSP00000332679
ENSP00000369050	ENSP00000320401	ENSP00000358851	ENSP00000251810	ENSP00000358727
ENSP00000342007	ENSP00000334276	ENSP00000256593	ENSP00000298556	ENSP00000381607
ENSP00000362249				
